# Endolithic algae influence the skeletal microstructure and porosity of reef-building corals

**DOI:** 10.1038/s41598-025-99374-1

**Published:** 2025-07-24

**Authors:** Edwin S. Uribe, Amalia Murgueitio, Carlos E. Gómez, Alberto Acosta, Juan A. Sánchez

**Affiliations:** 1 Laboratorio de Biología Molecular Marina-BIOMMAR, Departamento de Ciencias Biológicas, Facultad de Ciencias, Universidad de los Andes, Bogotá, D.C., Colombia; 2 Departamento de Biología, Facultad de ciencias,Pontificia Universidad Javeriana, UNESIS (Unidad de Ecología y Sistemática), Bogotá, D.C Colombia; 3 Department of Animal Ecology and Systematics, Justus Liebig University Giessen, Giessen, Germany

**Keywords:** Intracolonial analysis, Coral skeleton microenvironments, Coral-reef porosity, Microboring, micro-CT, *Porites* spp., Ecology, Environmental sciences, Ocean sciences

## Abstract

Coral skeletons provide habitat for a euendolithic community, forming a pigmented band within the skeleton, where *Ostreobium* is often a dominant group. Euendoliths actively penetrate live coral skeletons, but how they use and modify skeletal structure is not properly understood. This study explores the microstructural characteristics of skeletal microenvironments through a micro-CT technique that analyzes the “footprint” of the euendolithic community on the porosity of coral skeleton. We compared three *Porites* species samples, all of which exhibited a pigmented green band, based on the percentage of the relative volume of microporosity, macroporosity, total porosity, and solid volume fraction of calcium carbonate (CaCO_3_) among three distinct zones within the coral colony: coral tissue, the green band (characterized by the eundolithic community) and the bare skeletal region. We found a significant increase in microporosity within the green band, while the opposite occurs for macroporosity that decreased within this zone, for all analyzed species. We describe a model to explain the porosity gradient along the vertical axis for *Porites* coral colonies and suggests that within the “green band” microenvironment, the metabolic activity of the community is the responsible for this pattern. These findings suggest potential beneficial roles of the euendolithic community within coral holobionts, including microporosity filling via re-mineralization and mitigation of macroerosion.

**Supplementary Information:**

The online version contains supplementary material available at 10.1038/s41598-025-99374-1.

## Introduction

There has been growing interest in understanding the ecological role of microborers in coral skeletons. For instance, microbioerosion has been identified as the primary agent of bioerosion during the initial stages of colonizing dead coral substrates^1^, with erosion rates reaching up to 1 kg m^−2^ year^2,3^, where the endolithic community is primarily responsible for this process^4^. Coral skeletons provide vital niches for a diverse range of microorganisms, including algae, fungi, sponges, bacteria, viruses, and archaea, which vary in response to physical and chemical gradients such as oxygen, light, pH, and porosity^4–7^. Endolithic communities can create distinct pigmented bands beneath live coral tissue, visible in cross-sectional cuts of the skeleton^8,9^. While these bands are often green, depending on the composition of the endolithic community, other color bands—including black, gray-black, gray-green, yellow-green, brown, and pink—have been reported^7,10–13^. It has been observed that algae from the genus *Ostreobium* (Chlorophyta: Ulvophyceae: Bryopsidales) are the dominant microeukaryote group in the endolithic community of coral skeletons and other marine substrates^14^. *Ostreobium* spp. are often considered responsible for the formation of green bands^15–18^. However, studies on *Isopora palifera* (Lamarck, 1816) from Taiwan have identified green sulfur bacteria (*Prosthecochloris* spp.) as another potential source of green pigmentation in bands^12^. While recent research has focused on *Ostreobium* spp., there are still many gaps in our understanding of the biology of the endolithic community and their intricate relationships with corals^9^.

The ecological role of *Ostreobium* spp. within the coral holobiont emphasizes both beneficial and potentially harmful interactions for the host. On one hand, *Ostreobium* is thought to engage in mutualistic symbiosis with corals, transferring photo-assimilates and receiving shelter in return^17,19^. Likewise, these algae can offer an alternative source of energy to bleached corals (with live polyps), facilitating recovery by reducing light stress^6,18,20^. On the other hand, *Ostreobium* significantly contributes to microbioerosion in dead coral skeletons, dissolving up to 20% of deposited CaCO_3_^2,3^. As other euendoliths, it actively penetrates live coral skeletons, potentially weakening them and increasing vulnerability to mechanical damage^21^. Nevertheless, the balance of beneficial and detrimental roles of boring microalgae, as well as the exact characteristics of the microenvironments they use or create are not fully understood, and are still a matter of active research^4,9,22^.

Micro-CT (X-ray microtomography) porosity analysis is proposed as an innovative tool for studying micro and macrobioerosion in corals^23–25^. This technique utilizes X-ray beams to scan the three-dimensional structure of coral colonies at a micron-scale resolution^26^. CaCO_3_’s X-ray attenuation properties distinguish the solid coral skeleton from its macro and micropores, facilitating quantitative porosity analysis^24,27^. In contrast with other techniques, micro-CT offers several advantages, including 3D mapping, high resolution, non-destructive sample handling, and versatile scale assessments^25,28^. There are several studies utilizing micro-CT to analyze the porosity microenvironments of stony coral colonies^23,25,29^, but this technique has yet to be applied for studying the impact of the green band on coral skeleton porosity.

Corals with massive growth, such as *Porites* genus, serve as excellent models for studying intra-colonial microenvironments^3–5,15^. These growth forms usually stratify into different zones along a vertical profile, ranging from the outermost part of the colony (the polyps or living tissue zone) to the base, where the bare skeleton is located. However, since in many cases these skeletons exhibit a pigmented band containing *Ostreobium* spp^30,31^ and other endolithic microorganisms (e.g., cyanobacteria, fungi and green algae), this band can be delineated as a third zone. These three zones (coral tissue, pigmented band and skeleton) have been proposed and used in various studies, revealing significant changes in physicochemical variables along this vertical gradient^6,32^. For instance, some studies have identified microenvironmental gradients, with higher oxygen levels and pH in green bands and a decreasing pattern toward the base of the skeleton^5,21,33^. These gradients can be influenced by various factors, such as light availability, nutrient availability, pCO_2_, and water flow^21^.

Colombia has two oceanic basins (Pacific and Caribbean) that exhibit a wide variety of marine environments with contrasting characteristics. In the Caribbean Sea, high impact anthropogenic activities have significantly altered the natural conditions of coral ecosystems^34,35^. Conversely, in the Pacific, anthropogenic impacts have been lower, but environmental conditions are naturally more variable and abrupt, including lower alkalinity and carbonate saturation conditions and temperature changes due to ENSO^35,36^. In this context, Caribbean reefs are geographically more widespread, with broad bathymetric ranges with a wide diversity of coral species, but generally have low coral cover in most locations^35,37^. In contrast, the Colombian Pacific hosts coral reefs with a more limited geographical distribution and bathymetric range, featuring less diverse reef-forming assemblages, but a generally high coral cover^38,39^. Despite these known differences, exploration of potential variations in the intra-colonial physical structure of corals in both Colombian regions remains uncharted.

Given the ecological and economic significance of coral ecosystems^40^ and the context of accelerated coral reef degradation^41^, this study addresses the previously identified knowledge gaps that hinder the understanding of intracolonial microenvironments and the role of the endolithic community in coral bioerosion. Using the micro-CT technique, we characterize and compare the porosity of the internal skeletal areas of *Porites lobata* (Dana, 1846) and *P. panamensis* (Verrill, 1866) from the Colombian Pacific and *P. astreoides* (Lamarck, 1816) from the Caribbean. Specifically, the objective was to compare the vertical porosity profile of skeletal fragments, to determine whether these patterns vary with interspecific factors like location and coral species and finally to propose a model describing intracolonial variation. Additionally, Scanning Electron Microscopy (SEM) was employed to explore the morphology of pores and traces of bioerosion in these coral skeletal fragments. Given that our study focused on comparing intracolonial microenvironments, we did not perform a taxonomic characterization of the endolithic community. Consequently, we employ the broader functional term “endolithic community” to emphasize its collective bioeroding influence on skeletal porosity, rather than referring to individual taxonomic groups. However, all studied coral samples exhibited a visible green band, and previous work has confirmed the diversity of *Ostreobium* clades within the same biological collection from the species from this study (obtained from the same locations)^42^.

## Results

**Fig. 1:**
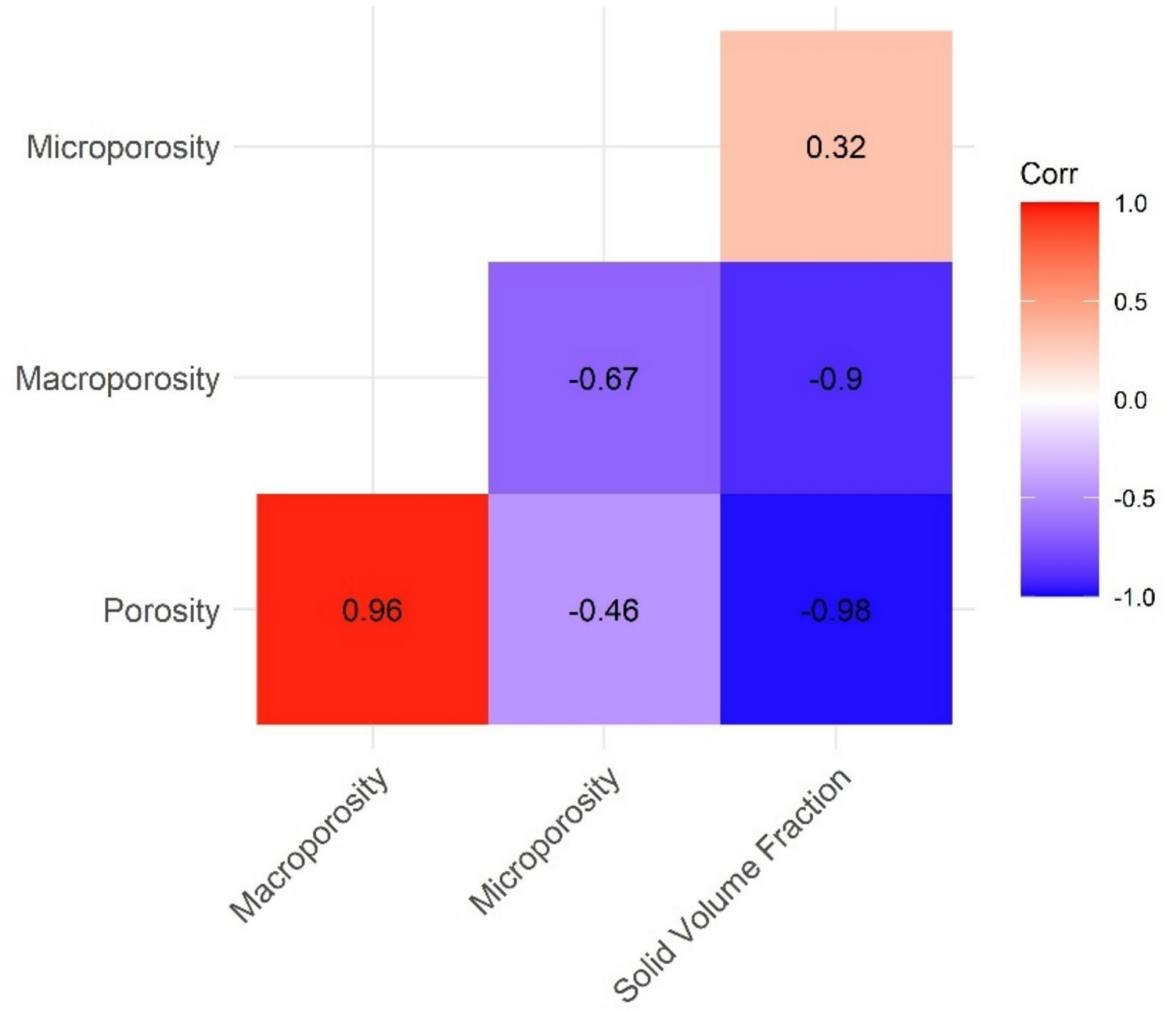
Intra-colonial scale Spearman correlations. Groups between response variables in the vertical profile (Z) of the fragments. All correlations were significant with *p*-value < 0.001.

Clear patterns were identified for microporosity, macroporosity, solid volume fraction, and total porosity, at different scales of analysis. At the intra-colonial scale, microporosity and macroporosity proved to be more relevant. Conversely, in the inter-specific scale, which involves the comparison between species and geographical locations, the variables solid volume fraction and total porosity gained more significance (Fig. 1).

### Intra-colonial porosity

**Fig. 2:**
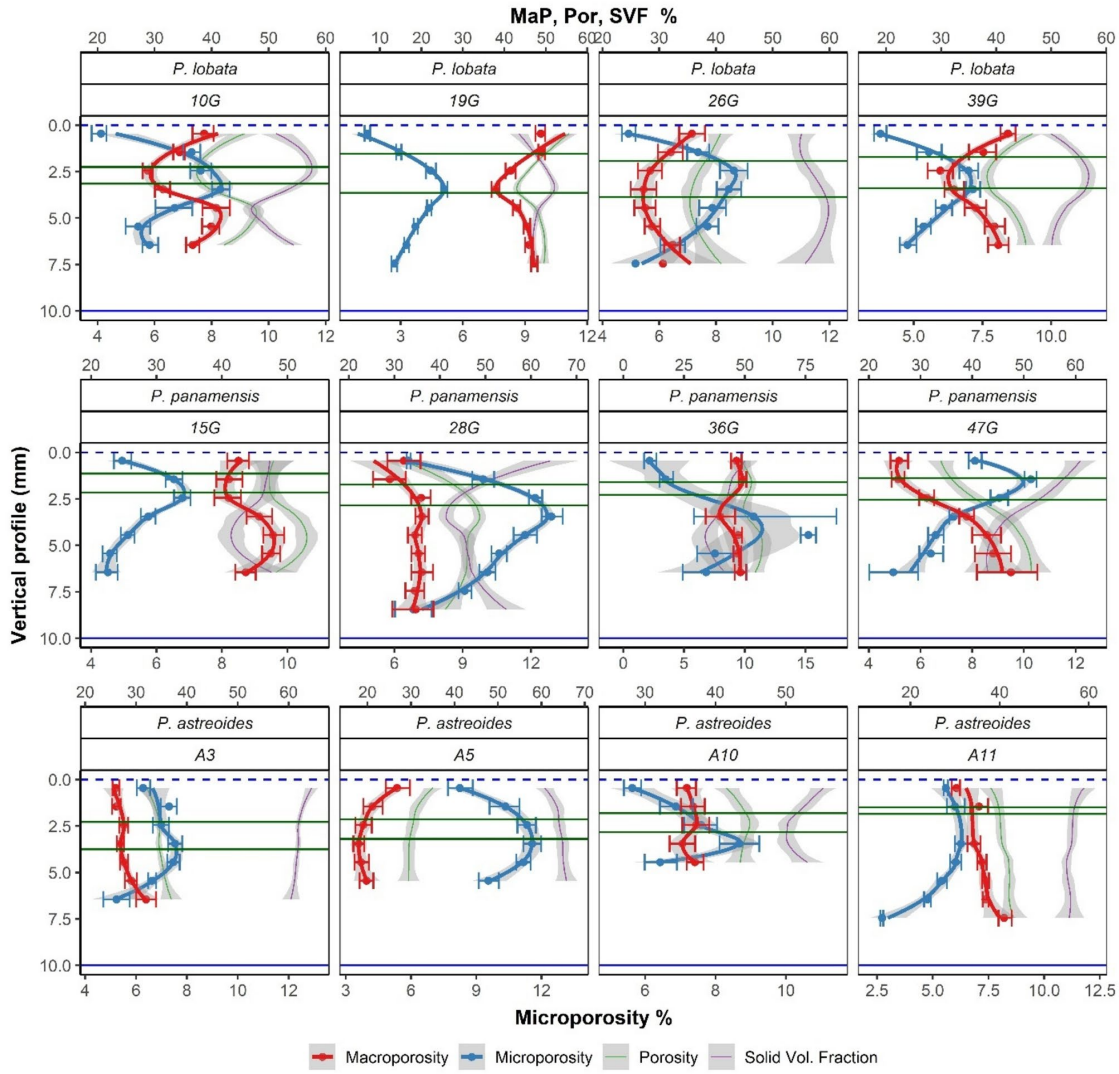
Vertical profile and colony comparison for the percentage (%) micro and macroporosity (MaP) (*n* = 12), total porosity (Por) and solid volume fraction (SVF). Data (points) represent average (± S.E.) for each sampled vertical coordinate, from the apical regions of the living tissue (vertical profile = 0 mm, dashed horizontal blue line, near the polyps) to the basal zone of the skeleton for each fragment (vertical profile = 10 mm, solid blue line and the green band location between them (solid green horizontal lines extracted from Table 1). Curves fitted from LOESS (local regression) and shades represent confidence intervals of the regressions.

The average microporosity across fragments indicates that micropores represent less than 11% of the total volume, while macroporosity accounts for up to 47% (Table 1). The mean solid volume fraction (overall 52%) exceeded total porosity (overall 42.7%) for most fragments (except for 15G and 28G), indicating that the percentage of CaCO_3_ is higher than the air spaces. Figure 2 shows the vertical profiles of fragments where the apical zone corresponds to the coral tissue at 0 mm, and the basal zone corresponds to the skeleton at approximately 10 mm (samples were cut at this size). However, the common total vertical length recorded by the porosity map (Z length Table 1) ranged between ~ 5 and ~ 9 mm because not all fragments had the same vertical length, due to variations in the cuts and segmentation results. Additionally, the observed green band locations varied between 1.1 mm and 3.9 mm along the vertical profile. Thus, this amplitude was used to distinguish the green band microenvironment from other zones within the fragment.

**Table 1:** Average ± standard errors (s.e.m) for the calculated response variables (microporosity - MiP, macroporosity - MaP, total porosity – total porosity & solid volume fraction - ) of the selected coral skeletal fragments: green band width (GB) in the vertical profile.

ID	Depth (m)	Species	Location	Z length (mm)	GB (mm)	MiP%	MaP%	TP%	SVF%
15G	12	P	Pacific	7	1.1–2.2	5.7 ± 0.08	44.4 ± 0.43	50.1 ± 0.40	44.2 ± 0.46
28G	12	P	Pacific	9	1.7–2.9	10.9 ± 0.12	34.9 ± 0.39	45.8 ± 0.40	46.1 ± 0.51
36G	12	P	Pacific	7	1.6–2.3	5.7 ± 0.74	47.0 ± 0.80	52.7 ± 0.69	42.6 ± 1.14
47G	12	P	Pacific	7	1.4–2.6	7.9 ± 0.12	35.6 ± 0.71	43.5 ± 0.62	50.3 ± 0.68
10G	12	L	Pacific	7	2.3–3.2	6.8 ± 0.11	35.5 ± 0.43	42.3 ± 0.37	53.1 ± 0.39
19G	12	L	Pacific	8	1.5–3.6	3.9 ± 0.06	44.0 ± 0.28	47.9 ± 0.24	48.5 ± 0.23
26G	12	L	Pacific	8	1.9–3.9	7.6 ± 0.11	29.8 ± 0.43	37.4 ± 0.38	57.5 ± 0.42
39G	12	L	Pacific	7	1.7–3.4	5.7 ± 0.09	36.8 ± 0.46	42.5 ± 0.38	52.9 ± 0.39
A10	35	A	Caribbean	5	1.8–2.8	7.3 ± 0.14	36.5 ± 0.43	43.8 ± 0.35	51.8 ± 0.38
A11	35	A	Caribbean	7	1.5–1.9	5.8 ± 0.09	35.5 ± 0.33	41.3 ± 0.28	55.7 ± 0.29
A3	9	A	Caribbean	7	2.3–3.8	7.2 ± 0.07	27.5 ± 0.21	34.6 ± 0.17	61.9 ± 0.14
A5	10	A	Caribbean	6	2.2–3.2	10.7 ± 0.14	20.1 ± 0.47	30.8 ± 0.37	64.0 ± 0.36
Overall					1.1–3.9	7.1 ± 0.05	35.6 ± 0.18	42.7 ± 0.15	52.2 ± 0.17

Notably, the percentages provided here are averages, thus, the sum of total porosity and solid volume fraction does not necessarily equal 100%. P = *Porites Panamensis;* L = *P. lobata*; A = *P. Astreoides.*

Repeated patterns of both variables (microporosity and macroporosity) were observed along the vertical axis of the 12 analyzed fragments (Fig. 2). The % of microporosity exhibits a monomodal curve with microporosity peaks (zones of high microporosity) related to the observed green band and an asymmetric shape towards the apical regions of the fragment. Conversely, macroporosity curves tend to decrease in the areas near the green band for the majority of the profiles. For the later variable, the monomodal behavior is not as evident as in microporosity, but the asymmetry of the valleys towards the apical regions is preserved. Regarding the vertical profiles of total porosity and solid volume fraction (Fig. 2), the curve shapes do not exhibit the prominent peaks and valleys observed in microporosity and macroporosity. However, a strong similarity between total porosity and macroporosity profiles is apparent, with some curves almost mirroring each other in shape (e.g., 47G *P. panamensis*, Fig. 1). Additionally, for solid volume fraction, the notable pattern observed is its inverse graphical correlation with macroporosity and total porosity.

The location (note that location ≠ magnitude) of peaks and valleys of the response variables in the vertical profile was correlated (Spearman correlation test) with the observed location of the green band in the stereoscope (solid horizontal green lines Fig. 1). A positive and significant correlation was found between microporosity peaks and the observed green band location (ρ = 0.66, *P* < 0.05) and between macroporosity valleys and the green band location (ρ = 0.72, *P* < 0.05). No significant differences were found between the locations of the green band and the locations of microporosity peaks (ANOVA: F = 0.4, *P* > 0.05) / macroporosity valleys (ANOVA: F = 0.9, *P* > 0.05). Spearman correlation across the fragment’s vertical profile (Fig. 1) detected an inverse relationship between microporosity and macroporosity (ρ=-0.67, *P* < 0.05), where microporosity increases as macroporosity decreases in the vertical profile (e.g., 10G *P. lobata* Fig. 1). The other variables exhibit the expected correlations, with total porosity being inversely correlated with solid volume fraction (ρ=-0.98, *P* < 0.05), and macroporosity positively correlated with total porosity (ρ = 0.96, *P* < 0.05). The macroporosity–total porosity correlation indicates that macropores, due to their large size, have the greatest influence on changes in the total porosity of the fragment, emphasizing the similarity between the vertical profiles of these two variables.

**Fig. 3:**
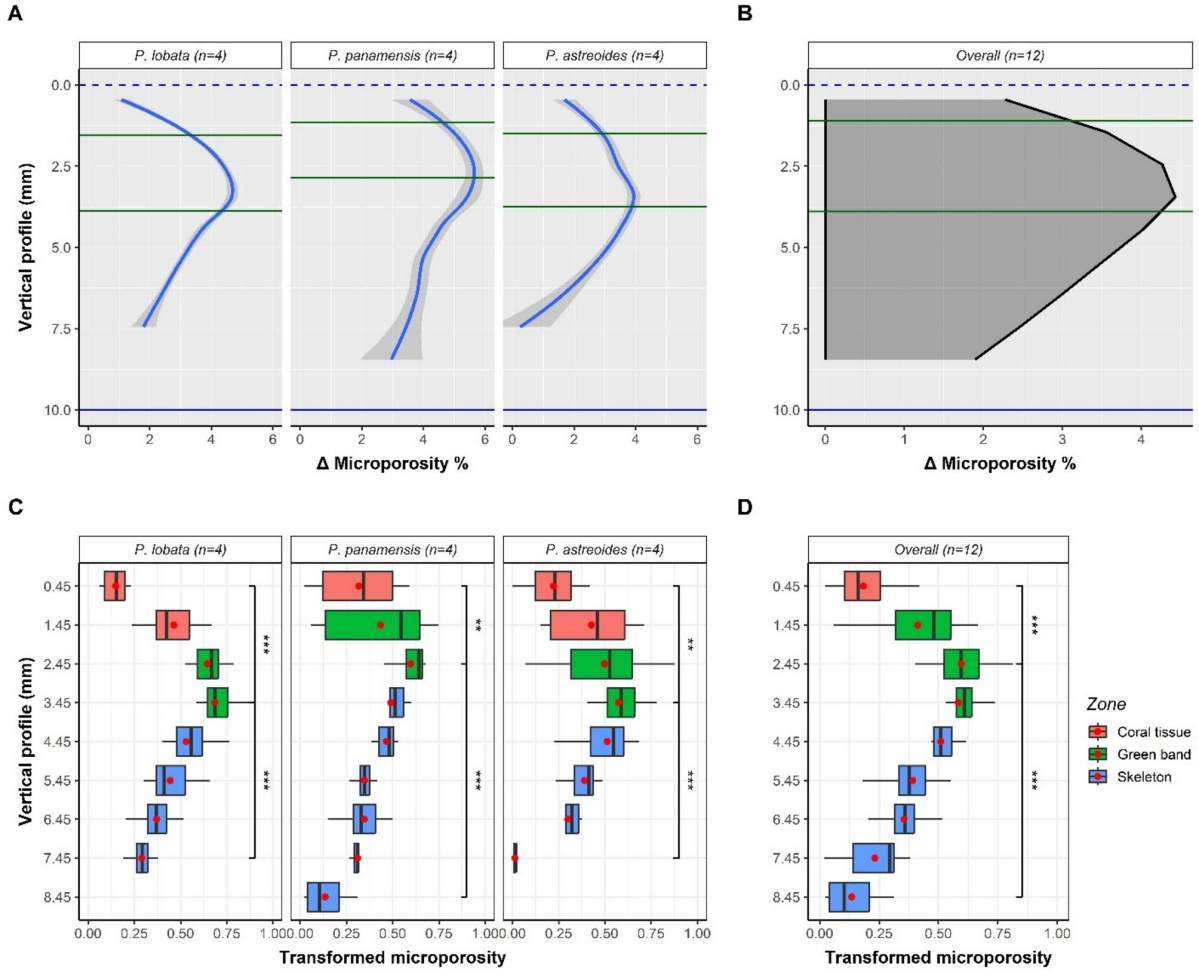
Intra-colonial variation overall and by species for % microporosity. Loess model by species (**A**) and overall (**B**) based on ∆ microporosity (Eq. 2). Boxplots by zones for species (**C**) and overall (**D**) derived from transformed microporosity (Eq. 2). Red dots and vertical lines inside the boxes represent the mean and median, respectively. Asterisks denote significant differences **(*p* < 0.01) and ***(*p* < 0.001).

Based on the Local Polynomial Regression Fitting (LOESS) model for microporosity for each species and an overall model (Fig. 3A, B), there is evidence for the influence of the endolithic community on coral skeletal structure, albeit some variation in peak height and amplitude. *Porites panamensis* exhibited the highest peak (∆max = 5.61%), followed by *P. lobata* (∆max = 4.65%), and *P. astreoides* (∆max = 3.93%). The overall model shows a maximum peak of 4.42% compared to the reference minimum value. The final pattern maintains the relationship observed, where there is an asymmetry towards the apices of the skeleton (vertical profile < 5 mm). Considering that the average microporosity of all fragments is ~ 7% (Table 1), the microporosity in the green band accounts for more than half of the average microporosity that can be present in colonies of these species. Significant differences were found for the three zones of coral fragments (Tukey HSD test) for all species and in the overall model. The microporosity of the green band zone is significantly different from the microporosity of the most extreme zones of living tissue and skeleton (supplementary material Table S1–S5) (Fig. 3C). Likewise, the single model maintains statistical differences between the three zones (Fig. 3D). These results indicate that the mean and median values found in the green band are significantly higher than those at the opposite ends of the other zones, thus, there are more similarities at the boundaries of the zones, and therefore, the changes between the different bands of the fragment are gradual across the vertical profile.

**Fig. 4:**
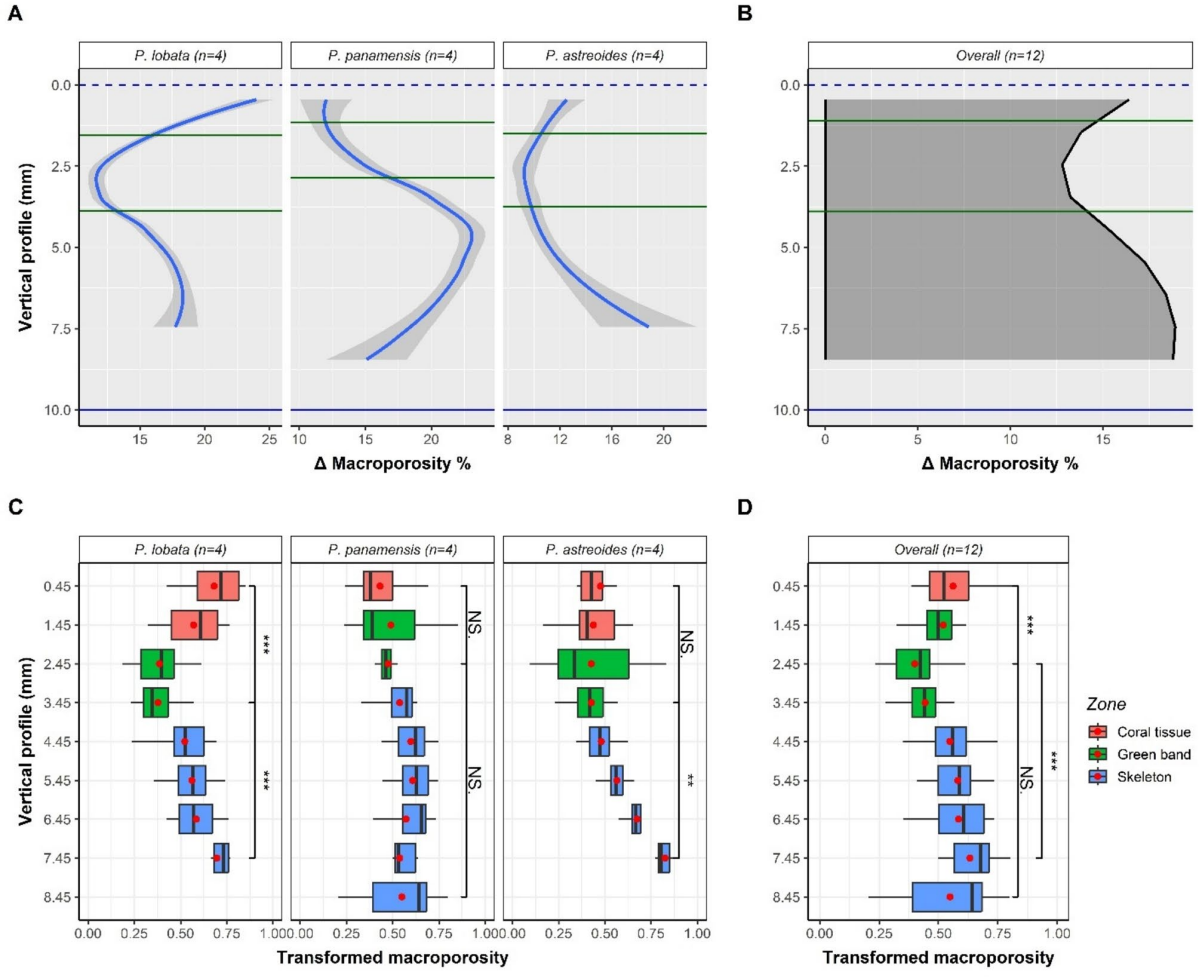
Intra-colonial variation overall and by species for % macroporosity. LOESS model by species (**A**) and overall (**B**) based on ∆ macroporosity (Eq. 1). Boxplots by zones for species (**C**) and overall (**D**) derived from transformed macroporosity (Eq. 1). Each red dot and vertical line inside the boxes refer to the mean and median, respectively. Asterisks denote significant differences ** (*p* < 0.01) and *** (*p* < 0.001), NS denotes no significant differences.

The LOESS model of macroporosity (Fig. 4A, B) shows low ∆ values forming valleys in the regions of the green band. The largest difference (∆max - ∆min) between the valleys of the green bands and the other zones of the vertical profile (skeleton and living tissue) was found in *P. lobata* (~ 12%) with a ∆min of ~ 12% and a ∆max of ~ 24%. In *P. panamensis* the difference was 10% with ∆min of ~ 12% and a ∆max of ~ 22%. Lastly, *P. astreoides* had a total difference of 9.5% with a ∆min and ∆max of 9.3% and 18.8%, respectively. The overall model showed a macroporosity valley with a ∆min of 12.8% in the green band and an increase in macroporosity (∆max of ~ 19%) towards the basal zones of the fragment, i.e., a total difference of 6.2%. Like the peaks of microporosity, the macroporosity valleys of the general and species-specific models are located towards the apical regions of the fragments (vertical profile < 5 mm).

The macroporosity in the different zones (Fig. 4C, D) shows the variations in the proposed LOESS models (Fig. 4A, B). For *P. panamensis*, the green band zone did not show significant differences for macroporosity compared to the other zones of the fragment (*P* > 0.05). Similarly, in *P. astreoides*, macroporosity values of the green band are not different from those of the living tissue, but they are different from the more basal zones (*P* < 0.01). Only for *P. lobata*, the significant differences of the green band are clear compared to the other zones (*P* < 0.001). The overall model maintains the variations seen at the species level; however, the most basal extreme of the fragment, at the 8.45 mm band in the vertical profile, are not different from the green band, mainly due to the high dispersion of values in this zone (whisker amplitude). In this case, it was compared with the previous band (7.45 mm), where significant differences are found (*P* < 0.001), as is also the case when comparing the green band to the living tissue (*P* < 0.001).

For solid volume fraction and total porosity, the intra-colonial results from both the overall and species models display inconsistencies. While the models exhibit some variations along the vertical profile, these variations do not consistently repeat in the same manner among the species models (supplementary material Figure S2 and S3). Consequently, the overall LOESS model exhibited a vertical profile that is predominantly linear, with no significant differences observed between the three microenvironments (*P* > 0.05). Despite the lack of consistent intra-specific variation, these variables are retained in the analysis because their significance becomes more apparent when discussing inter-specific differences.

**Fig. 5:**
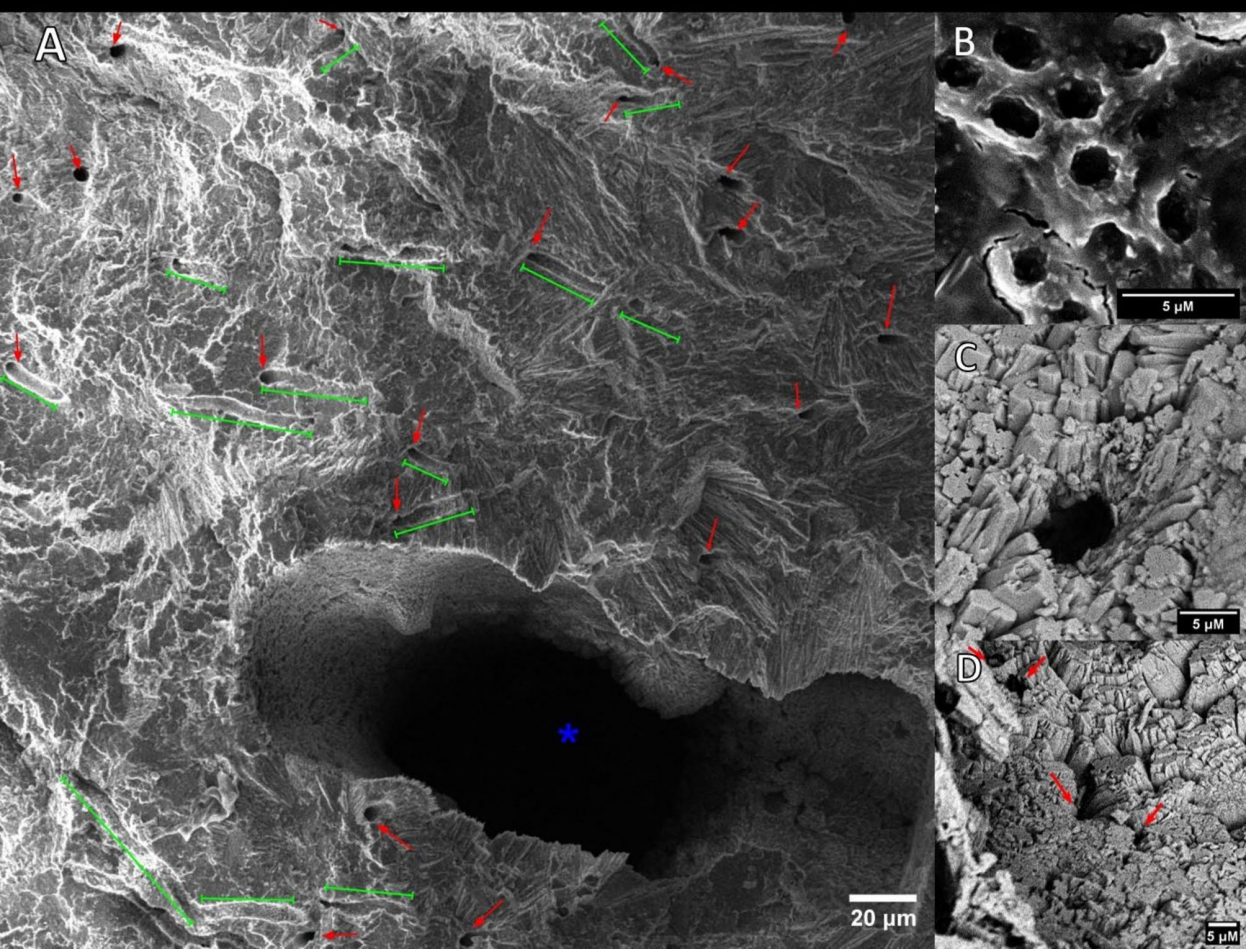
SEM Images showing a close-up of micropores (red arrows) and sinuous branching tracks (green lines) in comparison to macropores (blue asterisk) (**A**). Micropores observed in the green band zone (**B**), while in some areas, they are surrounded (**C**) or partially covered (**D**) by secondary precipitation crystals at the base of the skeleton.

The SEM images revealed traces of the endolithic community in the selected colonies and in different zones of the fragments. Figure 5A displays the typical structural differences between macro and micropores. The macropores are evident over the minimum resolution of micro-CT analysis (24.5 μm) and are even recognizable without any special equipment, as they comprise a significant proportion of the fragment volume. Conversely, the micropores, especially those related to the endolithic community, can be seen with a frequent diameter < 10 μm (Fig. 5A, B). Additionally, these are connected to tunnels that resemble the microborers’ filaments (Fig. 5A green bars) of the same diameter as the micropores. Even when the micro-CT analysis revealed that microporosity is higher in the green band, traces of these micro tunnels or sinuous branching tracks^36^ were seen qualitatively with SEM in all fragment zones, although less frequently in the coral tissue. The latter means that the euendolithic community is present along the vertical profile although in lower densities than the green band. However, one particularity is that these micropores were commonly surrounded by crystal-shaped structures in the skeleton zone (Fig. 5C) compared to a flatter surface in the green band micropores (Fig. 5B). In some cases, these crystals cover a large part of the pore space (Fig. 5D).

### Inter-specific porosity

**Fig. 6:**
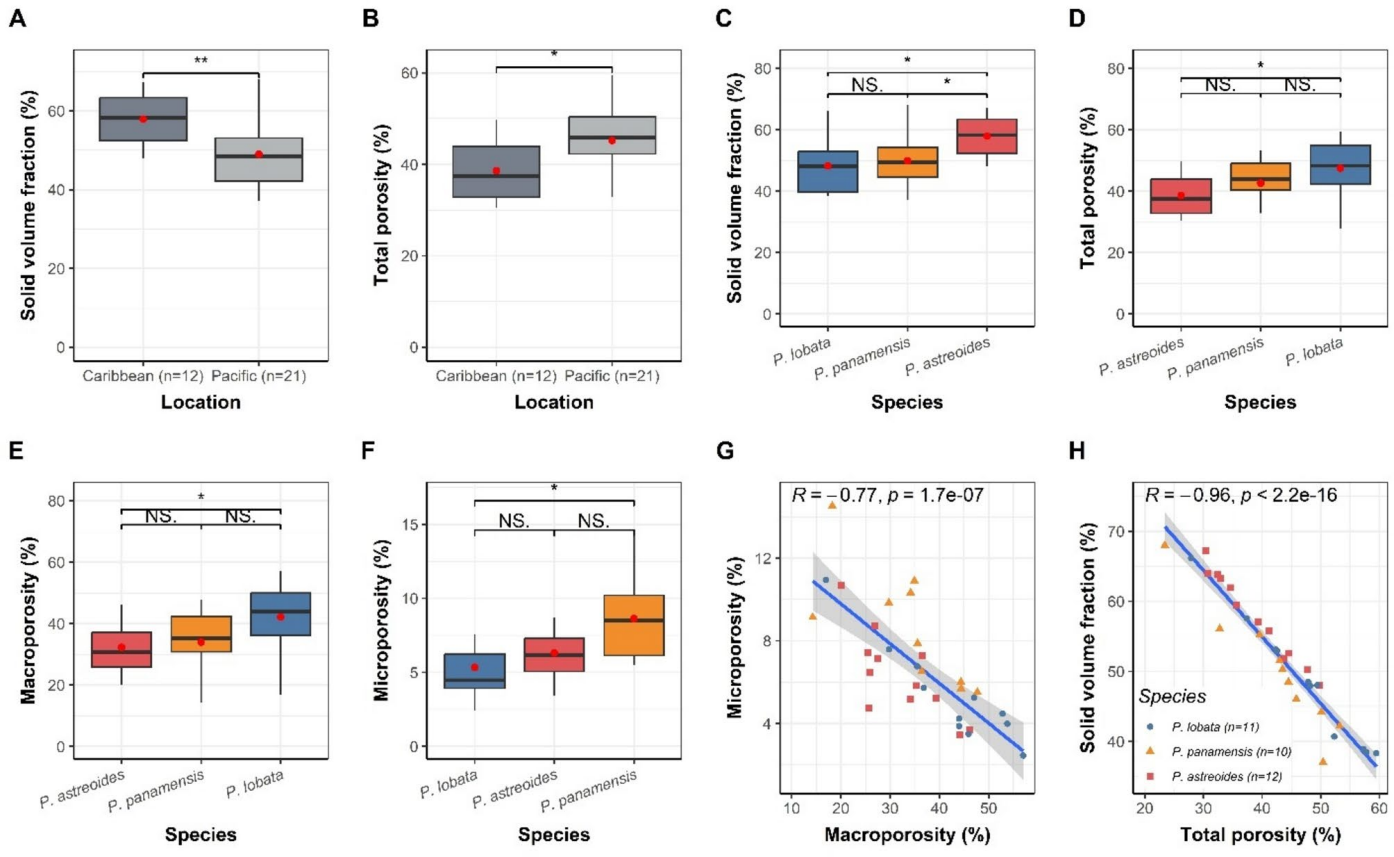
Percentage values of the inter-specific porosity (*n* = 30) for location comparisons in solid volume fraction (**A**) and total porosity (**B**) and species comparisons for solid volume fraction (**C**), total porosity (**D**), macroporosity (**E**), and microporosity (**F**). Correlations for different response variables (**G**, **H**). Asterisks denote significant differences * (*p* < 0.05), ** (*p* < 0.01).

Figure 6 shows the most relevant results conducted for the overall sample fragments (*n* = 30). Between locations (Caribbean and Pacific), significant differences (Kruskal-Wallis) were found only for solid volume fraction (Fig. 6A) (*P* < 0.01) and total porosity (Fig. 6B) (*P* < 0.05), indicating that fragments from the Caribbean have a higher solid volume (mean ~ 60%) and a lower total porosity volume (mean ~ 40%) than in the Pacific (mean solid volume fraction ~ 50% and total porosity ~ 45%). Although, no significant differences were detected between locations for macroporosity and microporosity (*P* > 0.05), the values indicate that, on average, Pacific fragments exhibit microporosity equivalent to 7.2% of the total colony volume, compared to 6.1% for the Caribbean. Concerning macroporosity, the mean value for the Pacific species (36.7%) is higher than the mean value of the Caribbean species (33%). These percentages align closely with the averages observed (microporosity ~ 7%, macroporosity ~ 36%) for the 12 selected fragments from the intra-colonial models.

The comparison between species shows significant differences for solid volume fraction (Fig. 6C) and microporosity (Fig. 6F), where *P. lobata* and *P. panamensis* were not statistically different to each other but different from *P. astreoides.* For the variable total porosity (Fig. 6D) and macroporosity (Fig. 6E), only *P. lobata* is statistically different from *P. astreoides*, which has the highest average value of solid volume fraction (58%), and the lowest values of total porosity (39%) and macroporosity (33%), followed by *P. panamensis* (solid volume fraction = 51%, total porosity = 41%, macroporosity = 32%) and *P. lobata* with the lowest values of solid volume fraction (49%) but the highest values of total porosity (46%) and macroporosity (40%). These results indicate that species from the Pacific are more porous than species from the Caribbean. However, in the case of microporosity (Fig. 6F), the differences between species do not align with the locations, as the two Pacific species are statistically different from each other (*P* < 0.05) but not different from *P. astreoides*. For microporosity, *P. panamensis* has the highest value (9.3%), followed by *P. astreoides* (6.1%), and *P. lobata* (5.3%).

The inverse relationship (Pearson correlation) between macroporosity and microporosity at the inter-specific level (Fig. 6G), where higher microporosity values coincide with lower macroporosity values (*R*=-0.77) are in accordance with the intra-colonial results (*R* = −0.6), indicating that it operates at different scales of analysis. The remaining results demonstrate the expected typical relationship, such as solid volume fraction being inversely correlated with total porosity (*R* = −0.96) (Fig. 6H) and the positive correlation between total porosity and macroporosity (*R* = 0.98) (supplementary material Figure S4).

## Discussion

Our findings revealed that the average microporosity values (~ 7%) were consistent with those reported for coral fragments of *Pocillopora damicornis* (Linnaeus, 1758) (~ 5–10%) and *Acropora aspera* (Dana, 1846) (~ 12–15%) under normal growth conditions^43^. However, the average macroporosity of 36% was higher than the ~ 15– 25% reported by Leggat et al.^43^. This might be attributed to the massive growth form of our coral skeletal fragments. Similarly, Krause et al.^44^ documented a higher total porosity of ~ 51% for *Porites*, exceeding the average total porosity of ~ 43% in our study. This observation aligns with previous findings indicating that massive growth species like *Porites* spp., tend to exhibit higher porosity (i.e., less dense skeletons) compared to branched and foliaceous species^45^. The observed differences could also be related to the life-history of our coral fragments, as species-specific biogeographical settings and associated environmental conditions can influence skeletal density and porosity^43^. Further comparative studies utilizing micro-CT porosity analysis across diverse coral species are needed to elucidate these relationships.

The position of the green band, indicative of mobile phototrophic community, was generally observed near the apical zones of the fragments, consistent with their light-seeking behavior. In our study, the green band in *Porites* species was located between 1 mm and 5 mm from the apical zone (living tissue), with a maximum thickness of 4 mm. While Kühl et al.^5^ reported a slightly deeper green band position in *Porites* (5–10 mm from the apical perimeter), their measured thicknesses (3–5 mm) align with our results. Similarly, other studies have documented green band positions ranging from 2 to 6 mm below the coral surface, with widths between 2 and 4 mm^32^, further supporting our observations. The asymmetry of porosity curves and the apical positions of the green band suggest a correlation between porosity peaks and valleys and areas of higher light intensity within the colony, as previously documented^16,21^. This alignment between asymmetric micro- and macroporosity curves within the green band is also consistent with reported patterns of intra-colonial gradients^4^.

The observed positive correlation and non-significant differences between the peak/valley’s locations of micro- and macroporosity, and the locations of the green band observed with the stereoscope method, indicate a consistent alignment between the two methodologies. However, stereoscopic measurements capture only surface features. While the green band thickness and position may differ between the surface and the interior of the coral skeleton, micro-CT provides a more precise 3-D analysis^24^. Furthermore, the coral skeleton itself acts as a record keeper, increasing the uncertainty regarding the exact position of the green band^46^. Traces of the green band, such as elevated microporosity, may persist in the skeleton even after the endolithic community has migrated, for example, as the colony grows and the community shifts towards areas of higher light intensity near the polyps^6^. Despite these potential sources of variations, our results show strong correlations between the positions and amplitudes (in mm) of the green band and the vertical profiles of micro- and macroporosities, supporting the use of micro-CT for delineating intra-colonial variation.

The consistent intra-colonial porosity patterns observed across sampled fragments and species, provide insights into the microenvironments of massive *Porites* species. The areas with a high concentration of the endolithic community (green band) exhibited significantly higher microporosity values compared to other microenvironments within coral skeleton. These observations align with other studies in which the metabolic activity within the green band leads to the formation of channels less than 10 μm in diameter^15,47^, classified as micropores in tomographic analysis^24,43^. Our micro-CT values revealed an average microporosity increase of approximately 4% within the green band. While this suggests a low overall influence of the endolithic community in the skeletal total porosity, it is important to note that this increase represents a substantial proportion of the total average microporosity (7%) observed in the analyzed fragments. Therefore, the green band represents the zone with the maximum achievable microporosity within analyzed colonies. This is particularly intriguing considering the results of a nanoindentation study in *Porites lutea* (Quoy & Gaimard, 1833) and *P. lobata*, which showed that hardness and Young’s modulus, a measure of material stiffness, were on average 1.4 times higher in the recently formed skeleton compared to the underlying skeleton^48^. Given that the green band likely occurs within a naturally harder and stiffer region of the coral, the localized increased in microporosity in the green band derives from endolithic microbioerosion rather than structural gradients that existed prior to microborer colonization.

While the coral skeleton preserves traces of past endolithic activity^46^, the observed decrease in microporosity below the green band suggests an active modification of the skeletal structure. Although microbioerosion by the endolithic community initially increases this value, why does not this increase persist below the green band? This process appears to be counteracted by secondary reprecipitation of CaCO_3_. SEM images (e.g., Fig. 5) reveal crystals characteristics of this secondary precipitation within the green band, supporting findings from other studies^44,49,50^. These studies demonstrate that endolithic algae, such as *Ostreobium*, can transport micro-eroded calcium from apical tips to the thallus base, where it re-precipitates as secondary aragonite along the pore edges. This “porosity filling” process, involves the cementation and compaction of crystals of less than 10 μm in width, in a form of early diagenesis, leading to observable changes within the coral skeleton as observed in our study and elsewhere^44,49,51^. This phenomenon likely contributes significantly to the gradual decrease in microporosity observed below the peak. Such evidence suggests new indirect relationship between endolithic community and coral host, supporting the mutualistic hypothesis proposed by Schlichter et al.^19^, and corroborated by more recent studies^3,6,52^. This hypothesis proposed that while endolithic activity can initially increase porosity, it also triggers secondary processes that ultimately enhance skeletal density and potentially benefit the coral holobiont.

Unexpectedly, the green band zone exhibits significantly lower macroporosity values compared to the coral tissue and skeleton. The minimum values observed across the entire vertical profile were consistently located within the green band, with an approximately 13% decrease, which represents a substantial mitigation of macroerosion, equivalent to approximately one-third of the typical macroporosity value (47%). In this context, our findings support new evidence of the role of the endolithic community to the coral skeletal matrix and coral-reef framework. The observed macroporosity valleys within the green band, suggest that the endolithic community, through direct and/or indirect mechanisms, actively influences the physical structure of the macropores within this skeletal region. Furthermore, the negative correlations between micro- and macroporosity, and to a lesser extent between micro- and total porosity, suggest a potential link between the increased microporosity and a reduction in both macropore size and overall skeletal porosity. This reduction in porosity could be attributed to the infilling of larger pores by secondary aragonite precipitation, as evidenced by the previously discussed SEM images and supporting literature.

While the specific mechanisms by which the green band is related to macropore reduction in the CaCO_3_ skeleton are beyond the scope of this article, existing research suggest a complex interplay between endolithic activity and skeletal porosity. This is achieved through a mechanism of enhanced photosynthetic activity within the green band, which captures CO_2_ increasing the pH, thereby diminishing chemical erosion by CaCO_3_ dissolution^5,52^. This pH increase potentially minimizes acidification-induced erosion, resulting in fewer air spaces and lower overall porosity within the skeleton. However, this apparent beneficial effect is offset by the increased metabolic activity and population density of the endolithic community that leads to greater microbioerosion in the form of tunnels, potentially causing skeletal damage (up to 7%)^21,44,49,50,52^. Essentially, the benefit of acidification mitigation also implies potential structural weakening through microboring. This suggest that sites experiencing lower pH conditions have corals with greater porosity probably due to more endolithic activity. For example, we found Pacific species like *P. lobata*, which typically experience different carbonate chemistry conditions including lower salinity and carbonate saturation states than the Caribbean Sea^36,53^, exhibited more macropores within the skeletons compared to Caribbean species like *P. astreoides* (Fig. 6B, E) as has been found in other studies^41^. This suggests a potential link between environmental variables, endolithic activity, and porosity, although further research is needed to confirm a direct cause-effect relationship.

Examining the precise effect of endolithic community on coral colonies using true colonial controls (fragments lacking a visible green band) presents significant challenges. Firstly, endolithic algae, such as *Ostreobium* spp., colonize coral skeletons early in the life cycle^47^. This early colonization makes it difficult to find coral fragments devoid of a green band for control groups, as colonization is likely throughout the coral’s lifespan^47^. Secondly, the absence of a visible green band does not guarantee the absence of endolithic activity. While chlorophyll pigmentation might fade over time (weak signal), the physical marks of microerosion, such as peaks in porosity and endolithic channels seen in SEM, persist^49^. This makes coral skeletons a valuable historical record of past endolithic activity^46^. As a point of comparison, rhodolite fragments were also analyzed in this study and exhibited no clear zonation or patterns in their vertical profiles, indicating that the observed patterns in the coral skeletons are indeed species-specific (supplementary material Figure S5).

The interspecific analysis further provides evidence for the relationship between coral species, geographic location and skeletal porosity, which helps to support the intra-colonial analysis. Although solid volume fraction, total porosity (total porosity), and macroporosity values support this interspecific pattern linked to acidification and geographic variation (Caribbean vs. Pacific), microporosity values surprisingly do not. This suggests that microporosity unlike the other porosity measures, is less influenced by locations or species. Instead, it is more sensitive at the already mentioned microenvironmental factors or gradients within the coral colony (intra-colonial scale as well as euendolithic community composition and activity. This is further supported by the observations that microporosity exhibits the most distinct patterns within the vertical profile rather than in interspecific comparisons.

In conclusion, our study provides insights into the relationship between endolithic community and skeletal porosity of massive *Porites* species, revealing distinct porosity patterns across three intra-colonial zones, suggesting a key role for endolithic metabolic activity in shaping the skeletal structure. Specifically, we observed a consistent increase in microporosity and a decrease in macroporosity within the green zone across all three studied species, regardless of their location, depth, or environment. This suggests a universal influence of endolithic communities on *Porites* skeletal structure. The green band is of particular interest, potentially mitigating total porosity by re-mineralization and porosity filling activity. Our findings highlight the influence of multiple intracolonial gradients playing a crucial role in the final distribution of porosity along the vertical profile. Therefore, we propose that endolithic communities exert a significant influence on the structural properties of the coral skeleton (i.e., macroporosity and total porosity), extending beyond the previously documented mutualistic benefits.

## Materials and methods

### Sample collection

Thirty (30) samples of coral skeletons corresponding to *Porites astreoides*,* P. lobata* and *P. panamensis* were selected from the Natural History Museum at Universidad de Los Andes (Bogota, Colombia). Samples were preserved as dry tissue without any chemical treatment. *Porites astreoides* samples (*n* = 10) were collected in 2018 from West View, San Andres Island (Caribbean Sea) (12° 31′ 15.45″ N, 81° 43′ 48.60″ W) (Supplementary material Figure S1) from a bathymetric range between 5 and 35 m, although statistical analyses confirmed no significant depth-related effect. *Porites lobata* (*n* = 10) and *P. panamensis* (*n* = 10) were collected in 2019 from Gorgona Island (Colombian Pacific) at a depth of 12 m (2° 9′ 2 4.19″ N , 78° 10′ 7.12″ W) (Supplementary material Figure S1). Using SCUBA, all samples were extracted with hammer and chisel, placed in plastic bags, and labelled for subsequent morphological identification based on the characteristics described in the literature^54,55^.

### Porosity analysis

#### Sample preparation

**Fig. 7:**
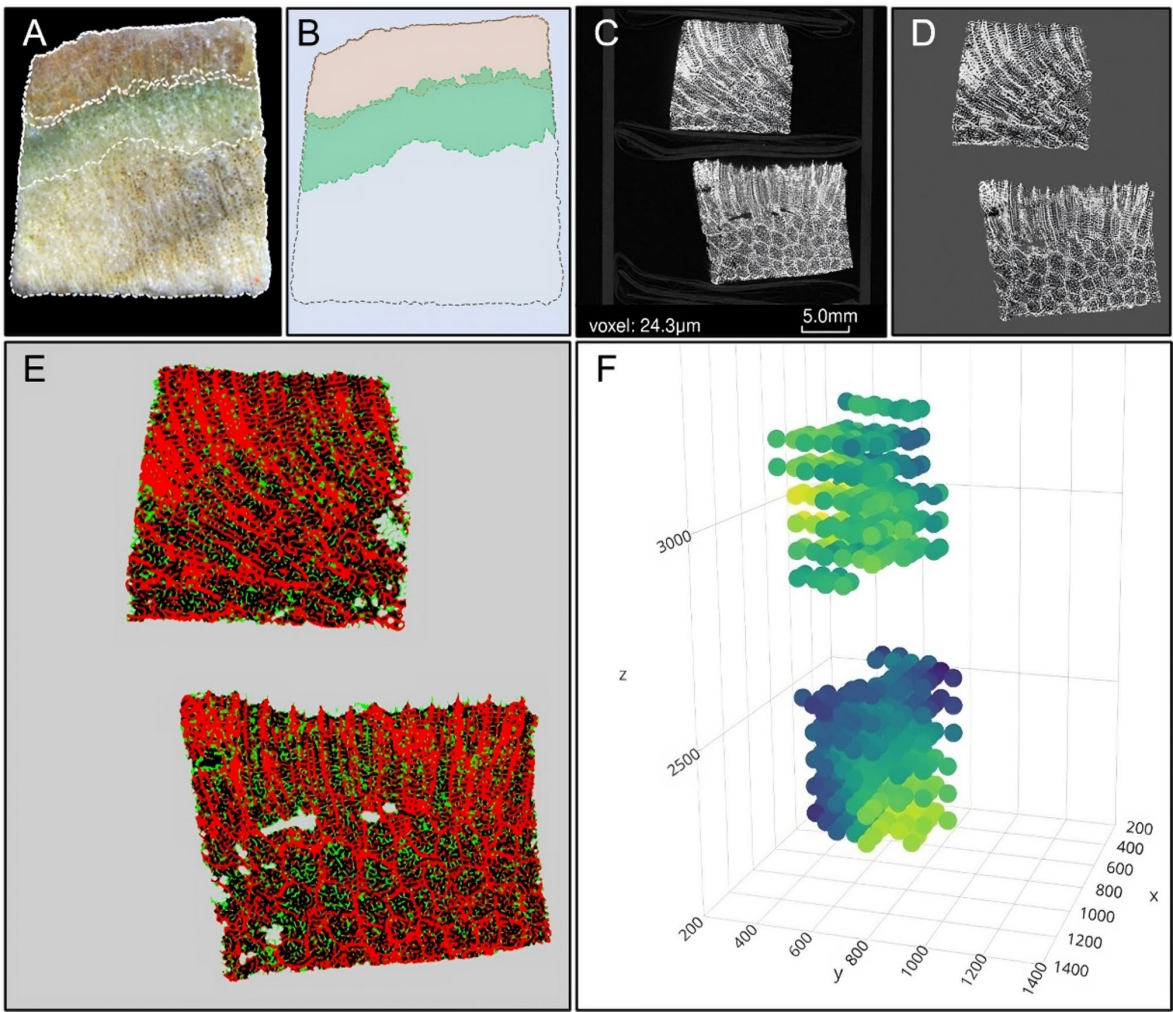
Image and Data Processing. Intra-colonial microenvironments viewed through the stereoscope (**A**) and measurements taken using ImageJ (**B**) (note the green zone where the euendolithic community is present within the coral skeleton and the three fragment microenvironments compared (polyps-pink, green band-green & skeleton-gray). Examples for two fragments include: raw tomographies (**C**), artifact-free images (**D**), segmentation results (**E**), and a porosity map depicting 3D axis coordinates (X, Y, Z) and total porosity values (dark tones means lower total porosity) (**F**).

Fragments of ~ 1 cm^3^ were cut using a Proxxon MBS 240/E diamond micro bandsaw. Photographs of all faces of the fragments were taken with Lumenera’s INFINITY1-1 M camera integrated with a JSZ6 stereoscope. A scale was included in each image to provide a reference for the fragment size. ImageJ software was employed for all measures such as polyp zone, green band and skeleton (Fig. 7). The samples were labeled and grouped in 50 ml Falcon tubes, with 5 to 6 fragments separated by a plastic layer. These samples were subsequently sent to the X-ray Micro-CT Laboratory at the Australian National University (ANU, Canberra, Australia) for processing with a Micro-CT scanner (CITES permit #46697).

#### Tomography processing

Each tomography produced a three-dimensional (3-D) NetCDF format (.nc) that had an original dimension of 1400 × 1400 × 3280 voxels (three-dimensional pixels) and a minimum resolution of 24.3 μm (Fig. 7C). Subsequently, 3-D images were uploaded to the WebMango platform (Australian National University), that facilitates tomographic modifications through a system of image filters. The sequence of this process involved three general steps: (1) masking filter (removal of artifacts such as the Falcon tube and fragment separators) (Fig. 7D), (2) segmentation filter (division of the image into phases or materials) (Fig. 7E), and (3) porosity analysis (quantitative estimation of in the skeletal structure) (Fig. 7F)^24^. Each voxel in the tomography belongs to one of four categories (colors). Red voxels denote areas where the skeleton is dense and solid. Black voxels represent air or embedded void spaces (macropores). Green areas represent an intermediate phase between the solid (red) and air (black) and they are technically termed micropores; those pores with sizes smaller than the tomographic resolution (24.3 μm), which influence tomographic intensity and are thus quantifiable^24^. Gray-colored voxels are referred to as mask, primarily indicating the volume within the tomography that does not belong to the colony. However, in specific cases, they may denote voids within the skeletal structure, which, due to their significant size, are connected to the exterior of the colony and are not considered as pores.

Using the volumes of the segmented image phases, a porosity analysis was performed to calculate four variables related to the total volume of the colony: (1) the relative volume (%) of microporosity, (2) macroporosity, (3) the total volume (%) of porosity, which is the sum of macro and microporosity percentages, and (4) the solid volume fraction, representing the relative volume (%) of the solid skeletal structure (CaCO_3_). Calculations were conducted along all three axes of the fragment-3D (X, Y, Z) at 0.9*0.9*0.9 mm distance intervals. Thus, this process generated a porosity map that replicated the original fragment’s shape, overlaying it with numerical values for the estimated variables (Fig. 7F). This approach facilitated the observation of changes or gradients in these four variables (microporosity, macroporosity, total porosity and solid volume fraction) along all three axes (X, Y, Z) of each fragment, enabling the assessment of skeletal variation, as well as the general differences among fragments from different species or locations in the Caribbean and the Pacific.

### Intra-colonial scale

The Z-axis is referred to as the vertical profile (Fig. 7F), enabling the examination of patterns within skeletal microenvironments. Notably, not all vertical profiles of the fragments were suitable for intraspecific analysis due to physical damage. Nevertheless, the fragments excluded from this analysis were still useful for interspecific comparisons (next section). Consequently, four fragments without physical damage were selected for each species (12 analyzed fragments). The zones observed in the stereoscope for the three microenvironments (coral tissue, green band & skeleton) along the vertical profile were assigned to each fragment based on measurements taken in ImageJ (see “Tomography processing” section). For every fragment, average porosity (± S.E.) was graphed for the different coordinates along the vertical profile.

#### Coordinates transformation

To compare skeletal variation among selected fragments, the coordinates of the three axes (X, Y, Z) of the porosity maps were transformed into a relative scale using Eq. 1 (Zjinorm). This transformation allowed for comparisons of fragment sizes and facilitated locating porosity data within the intra-colonial space with a common origin for all fragments. After this normalization, the scale was transformed into millimeters (mm) (Zjimm) (Eq. 1) based on a conversion factor (8 mm), representing the length of the largest porosity map sampled in the Z-axis, and a correction factor (0.45 mm), since each porosity point better represents the middle of the range sample interval of 0.9 mm (see “Tomography processing” section). For the Z-axis, the coordinate scale ranged between 0 and 10 mm for better visualization, where the limit of 10 referred to the most basal region of the skeleton, and the limit of 0 indicated the apical areas of the colony where living tissue (polyps) was situated. The same transformation was applied to the X and Y axes, but no clear porosity patterns were expected among fragments, as the most common stratification mentioned in the literature occurs along the vertical axis (Z-axis)^4,44^. However, some results for these two axes are briefly discussed in the present study. Equation (1) represents coordinate transformation to mm for each fragment along the Z axis (Zjimm):

1 $$\:{Zji}_{mm}=\:{(Zji}_{norm}*\:8\:mm)+0.45\:mm\:;{\:Zji}_{norm}=\:\frac{Zji-{Zji}_{min}\:}{\:{ZI}_{max}-\:{ZI}_{min}}$$

Where Zjinorm represents the normalized j-coordinate for fragment i, dependent on the minimum coordinate value of the i fragment (Zimin) as well as the maximum (ZImax) and minimum (ZImin) coordinates of the largest selected fragment I. It is important to note that the denominator is relative to the largest fragment (I) for standardized size comparisons among all selected fragments.

#### Variable transformation

The intra-colonial analysis for microporosity, macroporosity, total porosity and solid volume fraction involved transforming the values and constructing models to explain the vertical profile of the selected fragments. Two transformations were applied to the response variables, involving the calculation of deltas (∆) and data normalization (Eq. 2). The purpose of this equation is to preserve intra-colonial variation since statistics with raw (non-normalized) values tend to homogenize profile gradients, which would result in a vertical profile that becomes linear and fails to display the porosity fluctuations in the intra-colonial environments. Equation 2 represents transformation of response variables (microporosity, macroporosity) for Intra-specific analysis:

2 $$\:{Vji}_{norm}=\:\frac{\varDelta\:\:}{{\varDelta\:}_{max}}\:;\:\varDelta\:\:=\:Vji-{Vi}_{min}\:;\:\:{{\varDelta\:}_{max}\:=Vi}_{max}-{Vi}_{min\:}$$

Where, Vjinorm represents the normalized j-value of fragment i for the variable V. It is calculated as the delta between the unnormalized value (Vji) and its corresponding minimum value within the same fragment (Vimin) divided by the difference between the maximum value (Vimax) and the minimum value (Vimin).

### Inter-specific scale

The total number of samples analyzed (*n* = 30) were used to ensure a more robust statistical analysis. The response variables were not transformed, as intra-colonial gradients were not considered at this scale. Consequently, each fragment had only one porosity value, as opposed to a porosity map per fragment. To achieve this, the porosity map values of each fragment were averaged into a single value for microporosity, macroporosity, total porosity and solid volume fraction. At this inter-specific scale, the goal was to identify differences among all fragments between locations (Caribbean and Pacific) and species.

### Statistical analysis

#### Intra-colonial statistics

Local Polynomial Regression (LOESS) models were used to elucidate intra-colonial variation of the skeletal fragments, which consider local variation within a data series, based on a span of analyzed data points regarded as neighbors. The specific variation of intra-colonial porosity data points (e.g., Fig. 3F) was fitted into single curve across the axes of the fragments (X, Y, Z). Descriptive statistics, encompassing means and standard errors, were computed for the 12 samples. ANOVA and Tukey’s Honestly Significant Difference (HSD) test were performed to assess statistical differences between the three microenvironment zones (polyps, green band, and skeleton), following verification of normality and homoscedasticity assumptions. Finally, Spearman Correlation test was conducted between pairs of groups of response variables along the Z-axis of each fragment.

#### Inter-specific statistics

ANOVA and the Tukey’s Honestly Significant Difference (HSD) test for the response variables microporosity, macroporosity, total porosity and solid volume fraction were performed for species-level comparisons with balanced n-values, after verifying the assumptions of normality and homoscedasticity. For comparing locations, a non-parametric Kruskal-Wallis’s test was used, taking into account the imbalance in sample sizes between the Caribbean (*n* = 10) and the Pacific (*n* = 20), following inter-specific Spearman correlation test among response variables.

### Scanning electron microscopy

Additional fragments (*n* = 19) of ~ 1.5 cm long of the same coral colonies used for micro-CT analysis were obtained by fracturing them (hammer and chisel). The objective was to secure a vertical profile like the one illustrated in Fig. 2A, encompassing tissue, the green band, and the base. The samples underwent a hydrogen peroxide treatment to eliminate any remaining tissue in the colony, followed by rinsing with distilled water and overnight drying. To enhance conductivity, the unobserved faces of the fragments were covered with aluminum foil and affixed to a metallic surface using SEM conductive double-sided carbon tape. Additionally, a layer of gold was applied via vacuum coating prior to examination with a Tescan Vega 4 Scanning Electron Microscope. For each fragment, a general image was captured at 30X, followed by higher magnification close-ups in each of the three microenvironments (Coral tissue, green band & skeleton) at 100X, 500X, 1000X, 5000X, and 10000X to capture the porosity, micropore morphology, and bioerosion traces in the samples based on previous descriptions found in the literature^44,49^. Imaging was conducted at 10 keV.

## Electronic supplementary material

Below is the link to the electronic supplementary material.

Supplementary Material 1

## Data Availability

Some data are available in the main text or the supplementary materials. Raw data from Microtomography are all found in Figshare.com project 220825: DOI: 10.6084/m9.figshare.27041776 (https://figshare.com/account/projects/220825/articles/27041776).

## References

[1] Tribollet, A. & Golubic, S. Cross-shelf differences in the pattern and Pace of bioerosion of experimental carbonate substrates exposed for 3 years on the Northern great barrier reef, Australia. *Coral Reefs*. **24**, 422–434 (2005).

[2] Grange, J. S., Rybarczyk, H. & Tribollet, A. The three steps of the carbonate biogenic dissolution process by microborers in coral reefs (New Caledonia). *Environ. Sci. Pollut. Res.* **22**, 13625–13637 (2015). 10.1007/s11356-014-4069-z 25592911

[3] Tribollet, A. The boring microflora in modern coral reef ecosystems: A review of its roles. In *Current Developments in Bioerosion* (eds Wisshak, M. & Tapanila, L.) 67–94 (Springer, 2008). 10.1007/978-3-540-77598-0_4

[4] Ricci, F. et al. Beneath the surface: Community assembly and functions of the coral skeleton microbiome. *Microbiome* **7**, 159 (2019). 31831078 10.1186/s40168-019-0762-y PMC6909473

[5] Kühl, M., Holst, G., Larkum, A. W. D. & Ralph, P. J. Imaging of oxygen dynamics within the endolithic algal community of massive coral Porites lobata. *J. Phycol.* **44**, 541–550 (2008). 27041414 10.1111/j.1529-8817.2008.00506.x

[6] Galindo-Martínez, C. T. et al. The role of the endolithic *Alga ostreobium* spp. during coral bleaching recovery. *Sci. Rep.* **12**, 2977 (2022). 35194106 10.1038/s41598-022-07017-6 PMC8863988

[7] Yang, S. H. & Tang, S. L. Endolithic microbes in coral skeletons: Algae or bacteria? 43–53 (2019). 10.1007/978-94-024-1612-1_4

[8] Pasella, M. M., Lee, M. F. E., Marcelino, V. R., Willis, A. & Verbruggen, H. Ten ostreobium (ulvophyceae) strains from great barrier reef corals as a resource for algal endolith biology and genomics. *Phycologia* **61**, 452–458 (2022).

[9] Tandon, K. et al. Every refuge has its price: Ostreobium as a model for understanding how algae can live in rock and stay in business. *Semin. Cell Dev. Biol.* **134**, 27–36 (2023). 35341677 10.1016/j.semcdb.2022.03.010

[10] Priess, K., Le Campion-Alsumard, T., Golubic, S., Gadel, F. & Thomassin, B. A. Fungi in corals: Black bands and density-banding of *Porites lutea* and *P. lobata* skeleton. *Mar. Biol.* **136**, 19–27 (2000).

[11] Yamazaki, S. S., Nakamura, T. & Yamasaki, H. Photoprotective role of endolithic algae colonized in coral skeleton for the host photosynthesis. In *Photosynthesis. Energy from the Sun* (eds Allen, J. F., Gantt, E., Golbeck, J. H. & Osmond, B.) 1391–1395 (Springer, 2008). 10.1007/978-1-4020-6709-9_300

[12] Yang, S. H. et al. Prevalence of potential nitrogen-fixing, green sulfur bacteria in the skeleton of reef-building coral isopora palifera. *Limnol. Oceanogr.* **61**, 1078–1086 (2016).

[13] Yang, S. H. et al. Metagenomic, phylogenetic, and functional characterization of predominant endolithic green sulfur bacteria in the coral isopora palifera. *Microbiome* **7**, 3 (2019). 30609942 10.1186/s40168-018-0616-z PMC6320609

[14] Marcelino, V. R. & Verbruggen, H. Multi-marker metabarcoding of coral skeletons reveals a rich microbiome and diverse evolutionary origins of endolithic algae. *Sci. Rep.* **6**, 31508 (2016). 27545322 10.1038/srep31508 PMC4992875

[15] Le Campion-Alsumard, T., Golubic, S. & Hutchings, P. Microbial endoliths in skeletons of live and dead corals: *Porites lobata* (Moorea, French Polynesia). *Mar. Ecol. Prog Ser.* **117**, 149–157 (1995).

[16] Highsmith, R. C. Lime-boring algae in hermatypic coral skeletons. *J. Exp. Mar. Biol. Ecol.* **55**, 267–281 (1981).

[17] Del Campo, J., Pombert, J. F., Šlapeta, J., Larkum, A. & Keeling, P. J. The ‘other’ coral symbiont: *Ostreobium* diversity and distribution. *ISME J.* **11**, 296–299 (2017). 27420029 10.1038/ismej.2016.101 PMC5315466

[18] Iha, C. et al. Genomic adaptations to an endolithic lifestyle in the coral-associated *Alga ostreobium*. *Curr. Biol.* **31**, 1393–1402e5 (2021). 33548192 10.1016/j.cub.2021.01.018

[19] Schlichter, D., Zscharnack, B. & Krisch, H. Transfer of photoassimilates from endolithic algae to coral tissue. *Naturwissenschaften* **82**, 561–564 (1995).

[20] Fine, M. & Loya, Y. Endolithic algae: An alternative source of photoassimilates during coral bleaching. *Proc. R Soc. Lond. B* **269**, 1205–1210 (2002). 10.1098/rspb.2002.1983 PMC1691023 12065035

[21] Pernice, M. et al. Down to the bone: The role of overlooked endolithic microbiomes in reef coral health. *ISME J.* **14**, 325–334 (2020). 31690886 10.1038/s41396-019-0548-z PMC6976677

[22] Dievart, A. M., McQuaid, C. D., Zardi, G. I., Nicastro, K. R. & Froneman, P. W. Photoautotrophic euendoliths and their complex ecological effects in marine bioengineered ecosystems. *Diversity* **14**, 737 (2022).

[23] Tambutté, E. et al. Morphological plasticity of the coral skeleton under CO2-driven seawater acidification. *Nat. Commun.* **6**, 7368 (2015). 26067341 10.1038/ncomms8368 PMC4490415

[24] Fordyce, A. J. et al. Understanding decay in marine calcifiers: Micro-CT analysis of skeletal structures provides insight into the impacts of a changing climate in marine ecosystems. *Methods Ecol. Evol.* **11**, 1021–1041 (2020).

[25] Li, Y. et al. Micro-CT reconstruction reveals the colony pattern regulations of four dominant reef‐building corals. *Ecol. Evol.* **11**, 16266–16279 (2021). 34824826 10.1002/ece3.8308 PMC8601894

[26] Sentoku, A. et al. Regular budding modes in a zooxanthellate dendrophylliid urbinaria peltata (Scleractinia) revealed by X-ray CT imaging and three-dimensional reconstruction. *J. Morphol.* **276**, 1100–1108 (2015). 26129764 10.1002/jmor.20402

[27] Laforsch, C. et al. A precise and non-destructive method to calculate the surface area in living scleractinian corals using X-ray computed tomography and 3D modeling. *Coral Reefs* **27**, 811–820 (2008).

[28] Morales-Pinzón, A. et al. A semi-automatic method to extract canal pathways in 3D Micro-CT images of octocorals. *PLoS ONE* **9**, e85557 (2014). 24465599 10.1371/journal.pone.0085557 PMC3900427

[29] Roche, R. C., Abel, R. A., Johnson, K. G. & Perry, C. T. Quantification of porosity in *Acropora pulchra* (Brook 1891) using X-ray micro-computed tomography techniques. *J. Exp. Mar. Biol. Ecol.* **396**, 1–9 (2010).

[30] Halldal, P. Photosynthetic capacities and photosynthetic action spectra of endozoic algae of the massive coral Favia. *Biol. Bull.* **134**, 411–424 (1968).

[31] Jeffrey, S. W. Pigment composition of Siphonales algae in the brain coral Favia. *Biol. Bull.* **135**, 141–148 (1968).

[32] Magnusson, S., Fine, M. & Kühl, M. Light microclimate of endolithic phototrophs in the scleractinian corals *Montipora monasteriata* and *Porites cylindrica*. *Mar. Ecol. Prog Ser.* **332**, 119–128 (2007).

[33] Tribollet, A., Chauvin, A. & Cuet, P. Carbonate dissolution by reef microbialborers: A biogeological process producing alkalinity under different pCO2 conditions. *Facies* **65**, 9 (2019).

[34] Uribe, E. S. et al. A comprehensive threat analysis to support the red list of marine and coastal ecosystems of Colombia. *Front. Mar. Sci.* **9**, 962044 (2022).

[35] Uribe, E. S., Luna-Acosta, A. & Etter, A. Red list of ecosystems: Risk assessment of coral ecosystems in the Colombian Caribbean. *Ocean Coast. Manag.* **199**, 105416 (2021).

[36] Wizemann, A. et al. Rapid bioerosion in a tropical upwelling coral reef. *PLoS ONE* **13**, e0202887 (2018). 30208050 10.1371/journal.pone.0202887 PMC6135564

[37] Garzon-Ferreira, J. & Diaz, J. M. The Caribbean coral reefs of Colombia. In *Latin American Coral Reefs* (ed Cortés, J.) 275–301 (Elsevier, 2003).

[38] Zapata, F. A. & Vargas-Ángel, B. Corals and coral reefs of the Pacific coast of Colombia. In *Latin American Coral Reefs* (ed. Cortés, J.) 419–447 (Elsevier, 2003). 10.1016/B978-044451388-5/50019-9

[39] Díaz, J. M. et al. *Áreas Coralinas De Colombia* (INVEMAR, 2000).

[40] Costanza, R. et al. Changes in the global value of ecosystem services. *Glob. Environ. Change* **26**, 152–158 (2014).

[41] Fantazzini, P. et al. Gains and losses of coral skeletal porosity changes with ocean acidification acclimation. *Nat. Commun.* **6**, 7785 (2015). 26183259 10.1038/ncomms8785 PMC4518299

[42] Giraldo-Vaca, J. S. & Sánchez, J. A. Endolithic algae (Ostreobium) diversity in Porites corals at the Western Atlantic and tropical Eastern Pacific. *Mar. Ecol.* **45**, e12832 (2024).

[43] Leggat, W. P. et al. Rapid coral decay is associated with marine heatwave mortality events on reefs. *Curr. Biol.* **29**, 2723–2730e4 (2019). 31402301 10.1016/j.cub.2019.06.077

[44] Krause, S. et al. Endolithic algae affect modern coral carbonate morphology and chemistry. *Front. Earth Sci.* **7**, 304 (2019).

[45] Hughes, T. Skeletal density and growth form of corals. *Mar. Ecol. Prog Ser.* **35**, 259–266 (1987).

[46] Lartaud, F. et al. Growth patterns in long-lived coral species. In *Marine Animal Forests: The Ecology of Benthic Biodiversity Hotspots* (eds. Rossi, S., Bramanti, L., Gori, A. & Orejas Saco del Valle, C.) 1–32 (Springer, 2015). 10.1007/978-3-319-17001-5_15-1

[47] Massé, A., Domart-Coulon, I., Golubic, S., Duché, D. & Tribollet, A. Early skeletal colonization of the coral holobiont by the microboring *Ulvophyceae ostreobium* Sp. *Sci. Rep.* **8**, 2293 (2018). 29396559 10.1038/s41598-018-20196-5 PMC5797222

[48] Moynihan, M. A. et al. Environmental impact on the mechanical properties of Porites spp. Corals. *Coral Reefs* **40**, 701–717 (2021).

[49] Griffiths, N., Müller, W., Johnson, K. G. & Aguilera, O. A. Evaluation of the effect of diagenetic cements on element/Ca ratios in Aragonitic early miocene (~ 16 Ma) Caribbean corals: Implications for ‘deep-time’ palaeo-environmental reconstructions. *Palaeogeogr., Palaeoclimatol. Palaeoecol.* **369**, 185–200 (2013).

[50] Garcia-Pichel, F. Plausible mechanisms for the boring on carbonates by microbial phototrophs. *Sed. Geol.* **185**, 205–213 (2006).

[51] Ribaud-Laurenti, A., Hamelin, B., Montaggioni, L. & Cardinal, D. Diagenesis and its impact on Sr/Ca ratio in holocene Acropora corals. *Int. J. Earth Sci.* **90**, 438–451 (2001).

[52] Tribollet, A., Chauvin, A. & Cuet, P. Natural photosynthetic microboring communities produce alkalinity in seawater whereas Aragonite saturation state rises up to five. *Front. Earth Sci.* **10**, 894501 (2022).

[53] Manzello, D.P. et al. Poorly cemented coral reefs of the Eastern tropical Pacific: Possible insights into reef development in a high-CO2 world. *Proc. Natl. Acad. Sci.* **105** 10450–10455 (2008). 18663220 10.1073/pnas.0712167105 PMC2492517

[54] Reyes, J., Santodomingo, N. & Flórez, P. *Corales Escleractinios De Colombia* (INVEMAR, 2010).

[55] Maté, J. L., Brandt, M., Grassian, B. & Chiriboga, Á. Field guide to select Eastern Pacific corals and associated coral reef biota. In *Coral Reefs of the Eastern Tropical Pacific: Persistence and Loss in a Dynamic Environment* (eds Glynn, P. W., Manzello, D. P. & Enochs, I. C.) 593–637 (Springer, 2017). 10.1007/978-94-017-7499-4_22

