# Supplementary material for: Endolithic algae influence the skeletal microstructure and porosity of reef-building corals
Source: Sci Rep. 2025 Jul 24;15:26893. doi: 10.1038/s41598-025-99374-1 (PMC12289868; doi:10.1038/s41598-025-99374-1)

Supplementary material: The footprint of endolithic algae in shaping the skeletal structure of massive coral skeletons: insights into micro and macro-porosity

Edwin S. Uribe[1], Amalia Murgueitio[1], Carlos E. Gómez[1], Alberto Acosta[2], Juan A. Sánchez[1]

[1]Laboratorio de Biología Molecular Marina-BIOMMAR, Universidad de los Andes, Bogotá D.C, Colombia

[2]UNESIS (Unidad de Ecología y Sistemática), Departamento de Biología, Facultad de ciencias, Pontificia Universidad Javeriana, Bogotá, D.C., Colombia.

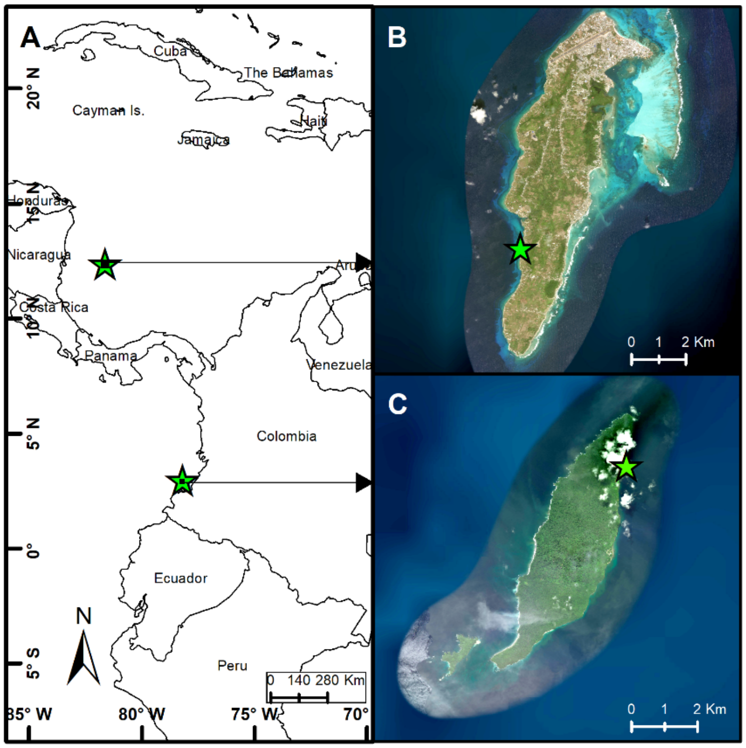

Figure S1. Study Area. Regional location of the sampling sites (green stars) (A) and close-ups of San Andrés Island - Caribbean (B) and Gorgona Island - Pacific (C)

Table S1. Complete Tukey HSD test of Microporosity for *P. panamensis* vertical bands.

Microporosity MiP *P. panamensis*				
Vertical bands (mm)	diff	lwr	upr	p adj
1.45-0.45	0.116	-0.090	0.322	0.687
2.45-0.45	0.280	0.054	0.505	0.005
3.45-0.45	0.173	-0.037	0.383	0.194
4.45-0.45	0.148	-0.066	0.363	0.413
5.45-0.45	0.030	-0.181	0.240	1.000
6.45-0.45	0.028	-0.191	0.248	1.000
7.45-0.45	-0.007	-0.249	0.235	1.000
8.45-0.45	-0.183	-0.436	0.071	0.354
2.45-1.45	0.163	-0.049	0.376	0.273
3.45-1.45	0.057	-0.140	0.253	0.991
4.45-1.45	0.032	-0.169	0.233	1.000
5.45-1.45	-0.087	-0.283	0.110	0.893
6.45-1.45	-0.088	-0.294	0.119	0.911
7.45-1.45	-0.123	-0.353	0.107	0.742
8.45-1.45	-0.299	-0.541	-0.057	0.005
3.45-2.45	-0.107	-0.323	0.110	0.817
4.45-2.45	-0.131	-0.352	0.089	0.618
5.45-2.45	-0.250	-0.466	-0.034	0.012
6.45-2.45	-0.251	-0.477	-0.026	0.018
7.45-2.45	-0.286	-0.534	-0.039	0.011
8.45-2.45	-0.462	-0.721	-0.204	0.000
4.45-3.45	-0.025	-0.229	0.180	1.000
5.45-3.45	-0.143	-0.344	0.057	0.367
6.45-3.45	-0.144	-0.355	0.066	0.421
7.45-3.45	-0.180	-0.413	0.054	0.270
8.45-3.45	-0.356	-0.601	-0.110	0.000
5.45-4.45	-0.119	-0.324	0.086	0.651
6.45-4.45	-0.120	-0.334	0.094	0.693
7.45-4.45	-0.155	-0.392	0.082	0.491
8.45-4.45	-0.331	-0.580	-0.082	0.002
6.45-5.45	-0.001	-0.211	0.209	1.000
7.45-5.45	-0.036	-0.270	0.197	1.000
8.45-5.45	-0.212	-0.458	0.033	0.144
7.45-6.45	-0.035	-0.277	0.207	1.000
8.45-6.45	-0.211	-0.465	0.042	0.180

8.45-7.45	-0.176	-0.449	0.097	0.511

Table S2. Complete Tukey HSD test of Macroporosity for *P. panamensis* vertical bands.

Macroporosity MaP *P. panamensis*				
Vertical bands (mm)	diff	lwr	upr	p adj
1.45-0.45	0.059	-0.156	0.273	0.994
2.45-0.45	0.044	-0.191	0.278	1.000
3.45-0.45	0.106	-0.112	0.325	0.826
4.45-0.45	0.166	-0.057	0.389	0.312
5.45-0.45	0.176	-0.042	0.394	0.216
6.45-0.45	0.141	-0.087	0.369	0.571
7.45-0.45	0.107	-0.145	0.358	0.912
8.45-0.45	0.117	-0.146	0.381	0.887
2.45-1.45	-0.015	-0.236	0.206	1.000
3.45-1.45	0.048	-0.156	0.252	0.998
4.45-1.45	0.107	-0.102	0.316	0.781
5.45-1.45	0.117	-0.087	0.322	0.661
6.45-1.45	0.082	-0.133	0.296	0.950
7.45-1.45	0.048	-0.191	0.287	0.999
8.45-1.45	0.059	-0.193	0.310	0.998
3.45-2.45	0.063	-0.162	0.288	0.993
4.45-2.45	0.122	-0.107	0.352	0.744
5.45-2.45	0.132	-0.093	0.357	0.632
6.45-2.45	0.097	-0.137	0.331	0.923
7.45-2.45	0.063	-0.194	0.320	0.997
8.45-2.45	0.074	-0.195	0.342	0.994
4.45-3.45	0.060	-0.153	0.272	0.993
5.45-3.45	0.070	-0.139	0.278	0.978
6.45-3.45	0.034	-0.184	0.253	1.000
7.45-3.45	0.000	-0.242	0.243	1.000
8.45-3.45	0.011	-0.244	0.266	1.000
5.45-4.45	0.010	-0.203	0.223	1.000
6.45-4.45	-0.025	-0.248	0.197	1.000
7.45-4.45	-0.059	-0.306	0.187	0.997
8.45-4.45	-0.049	-0.307	0.210	1.000
6.45-5.45	-0.035	-0.254	0.183	1.000

7.45-5.45	-0.069	-0.312	0.173	0.992
8.45-5.45	-0.059	-0.314	0.196	0.998
7.45-6.45	-0.034	-0.285	0.217	1.000
8.45-6.45	-0.023	-0.287	0.240	1.000
8.45-7.45	0.011	-0.273	0.294	1.000

Table S3. Complete Tukey HSD test of Microporosity for P. lobata vertical bands.

Micropososity MiP P. lobata				
Vertical bands (mm)	diff	lwr	upr	p adj
1.45-0.45	0.314	0.210	0.418	0.000
2.45-0.45	0.496	0.392	0.599	0.000
3.45-0.45	0.535	0.431	0.638	0.000
4.45-0.45	0.380	0.277	0.484	0.000
5.45-0.45	0.295	0.191	0.398	0.000
6.45-0.45	0.221	0.117	0.325	0.000
7.45-0.45	0.141	-0.007	0.288	0.074
2.45-1.45	0.182	0.081	0.282	0.000
3.45-1.45	0.221	0.120	0.321	0.000
4.45-1.45	0.066	-0.035	0.167	0.471
5.45-1.45	-0.020	-0.120	0.081	0.999
6.45-1.45	-0.093	-0.194	0.008	0.093
7.45-1.45	-0.174	-0.319	-0.028	0.008
3.45-2.45	0.039	-0.062	0.140	0.934
4.45-2.45	-0.116	-0.216	-0.015	0.013
5.45-2.45	-0.201	-0.302	-0.101	0.000
6.45-2.45	-0.275	-0.376	-0.174	0.000
7.45-2.45	-0.355	-0.501	-0.210	0.000
4.45-3.45	-0.155	-0.255	-0.054	0.000
5.45-3.45	-0.240	-0.341	-0.139	0.000
6.45-3.45	-0.314	-0.414	-0.213	0.000
7.45-3.45	-0.394	-0.539	-0.249	0.000
5.45-4.45	-0.086	-0.186	0.015	0.159
6.45-4.45	-0.159	-0.260	-0.059	0.000
7.45-4.45	-0.240	-0.385	-0.094	0.000
6.45-5.45	-0.074	-0.174	0.027	0.329
7.45-5.45	-0.154	-0.299	-0.009	0.030
7.45-6.45	-0.080	-0.226	0.065	0.684

Table S4. Complete Tukey HSD test of Macroporosity for *P. lobata* vertical bands.

Macropososity MaP P. lobata				
Vertical bands (mm)	diff	lwr	upr	p adj
1.45-0.45	-0.111	-0.238	0.016	0.135
2.45-0.45	-0.296	-0.423	-0.168	0.000
3.45-0.45	-0.303	-0.430	-0.175	0.000
4.45-0.45	-0.159	-0.286	-0.031	0.005
5.45-0.45	-0.120	-0.247	0.007	0.080
6.45-0.45	-0.096	-0.223	0.032	0.294
7.45-0.45	0.016	-0.165	0.197	1.000
2.45-1.45	-0.185	-0.308	-0.061	0.000
3.45-1.45	-0.192	-0.315	-0.068	0.000
4.45-1.45	-0.047	-0.171	0.076	0.936
5.45-1.45	-0.009	-0.133	0.115	1.000
6.45-1.45	0.016	-0.108	0.139	1.000
7.45-1.45	0.127	-0.051	0.306	0.362
3.45-2.45	-0.007	-0.131	0.117	1.000
4.45-2.45	0.137	0.013	0.261	0.019
5.45-2.45	0.176	0.052	0.299	0.001
6.45-2.45	0.200	0.076	0.324	0.000
7.45-2.45	0.312	0.133	0.490	0.000
4.45-3.45	0.144	0.021	0.268	0.011
5.45-3.45	0.183	0.059	0.306	0.000
6.45-3.45	0.207	0.083	0.331	0.000
7.45-3.45	0.319	0.140	0.497	0.000
5.45-4.45	0.039	-0.085	0.162	0.979
6.45-4.45	0.063	-0.061	0.187	0.770
7.45-4.45	0.175	-0.004	0.353	0.060
6.45-5.45	0.024	-0.099	0.148	0.999
7.45-5.45	0.136	-0.043	0.315	0.276
7.45-6.45	0.112	-0.067	0.290	0.535

Table S5. Complete Tukey HSD test of Microporosity for *P. astreoides* vertical bands.

Micropososity MiP P. astreoides				
Vertical bands (mm)	diff	lwr	upr	p adj
1.45-0.45	0.206	0.024	0.388	0.016
2.45-0.45	0.276	0.098	0.454	0.000
3.45-0.45	0.352	0.173	0.530	0.000

4.45-0.45	0.290	0.103	0.477	0.000
5.45-0.45	0.168	-0.029	0.365	0.155
6.45-0.45	0.078	-0.163	0.320	0.972
7.45-0.45	-0.209	-0.520	0.103	0.438
2.45-1.45	0.070	-0.091	0.231	0.877
3.45-1.45	0.146	-0.015	0.307	0.107
4.45-1.45	0.084	-0.087	0.255	0.790
5.45-1.45	-0.038	-0.220	0.144	0.998
6.45-1.45	-0.127	-0.357	0.102	0.674
7.45-1.45	-0.414	-0.717	-0.112	0.001
3.45-2.45	0.076	-0.081	0.232	0.808
4.45-2.45	0.014	-0.153	0.181	1.000
5.45-2.45	-0.108	-0.286	0.070	0.568
6.45-2.45	-0.198	-0.424	0.029	0.133
7.45-2.45	-0.485	-0.785	-0.185	0.000
4.45-3.45	-0.061	-0.228	0.105	0.946
5.45-3.45	-0.184	-0.362	-0.006	0.038
6.45-3.45	-0.273	-0.499	-0.047	0.007
7.45-3.45	-0.560	-0.860	-0.260	0.000
5.45-4.45	-0.122	-0.309	0.065	0.471
6.45-4.45	-0.212	-0.445	0.022	0.104
7.45-4.45	-0.499	-0.804	-0.193	0.000
6.45-5.45	-0.090	-0.331	0.152	0.944
7.45-5.45	-0.377	-0.688	-0.065	0.007
7.45-6.45	-0.287	-0.629	0.054	0.167

Table S6. Complete Tukey HSD test of Macroporosity for *P. astreoides* vertical bands.

Macropososity MaP *P. astreoides*				
Vertical bands (mm)	diff	lwr	upr	p adj
1.45-0.45	-0.039	-0.212	0.134	0.997
2.45-0.45	-0.050	-0.219	0.119	0.984
3.45-0.45	-0.050	-0.219	0.120	0.985
4.45-0.45	0.004	-0.174	0.181	1.000
5.45-0.45	0.088	-0.099	0.276	0.824
6.45-0.45	0.197	-0.032	0.426	0.146
7.45-0.45	0.349	0.053	0.645	0.010
2.45-1.45	-0.011	-0.164	0.142	1.000
3.45-1.45	-0.010	-0.163	0.143	1.000

4.45-1.45	0.043	-0.120	0.205	0.992
5.45-1.45	0.128	-0.045	0.300	0.311
6.45-1.45	0.236	0.019	0.454	0.024
7.45-1.45	0.388	0.101	0.675	0.002
3.45-2.45	0.001	-0.148	0.149	1.000
4.45-2.45	0.054	-0.105	0.212	0.965
5.45-2.45	0.139	-0.031	0.308	0.192
6.45-2.45	0.247	0.033	0.462	0.013
7.45-2.45	0.399	0.114	0.684	0.001
4.45-3.45	0.053	-0.105	0.211	0.967
5.45-3.45	0.138	-0.031	0.307	0.197
6.45-3.45	0.247	0.032	0.461	0.013
7.45-3.45	0.398	0.113	0.683	0.001
5.45-4.45	0.085	-0.093	0.262	0.816
6.45-4.45	0.193	-0.028	0.415	0.133
7.45-4.45	0.345	0.055	0.635	0.009
6.45-5.45	0.109	-0.121	0.338	0.821
7.45-5.45	0.260	-0.036	0.556	0.127
7.45-6.45	0.152	-0.172	0.476	0.831

Figure S2. Vertical profiles for Solid Volume Fraction (SVF).

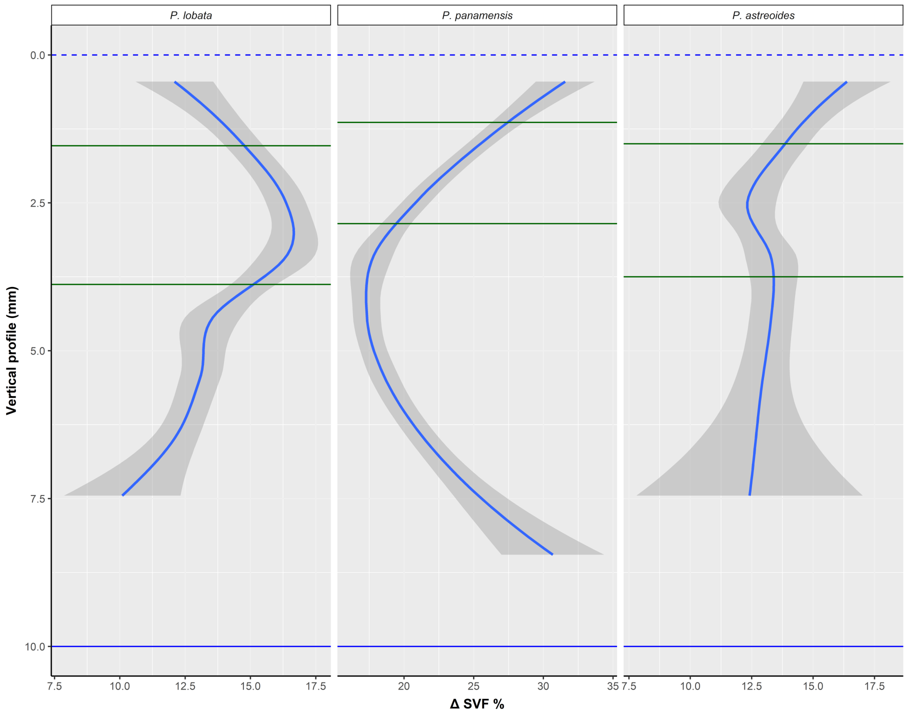

Figure S3. Vertical profiles for Total Porosity (TP)

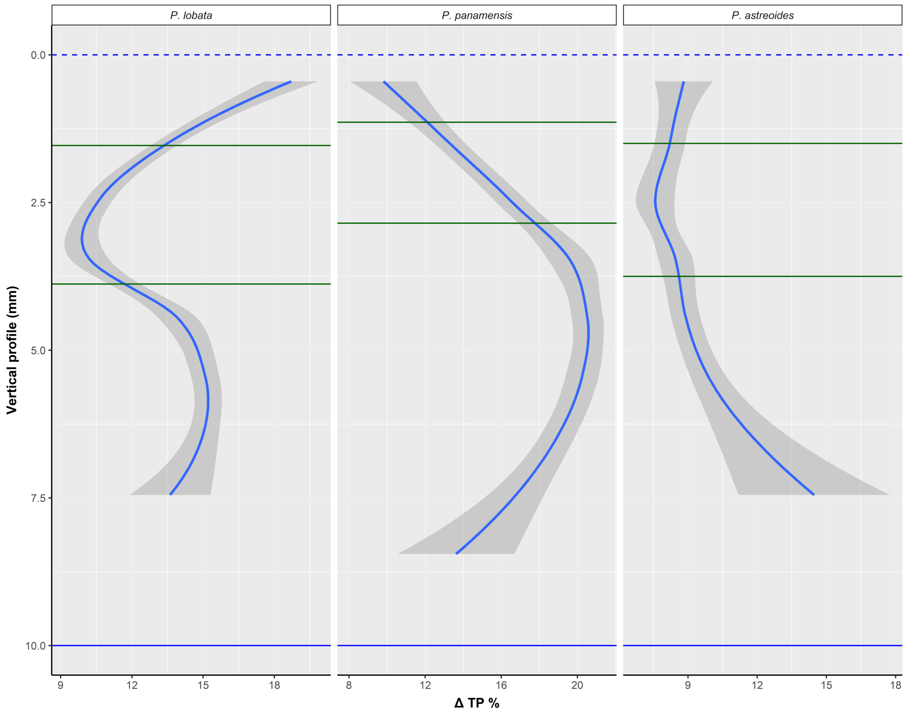

Figure S4. Solid Volume Fraction and Total Porosity expected correlation.

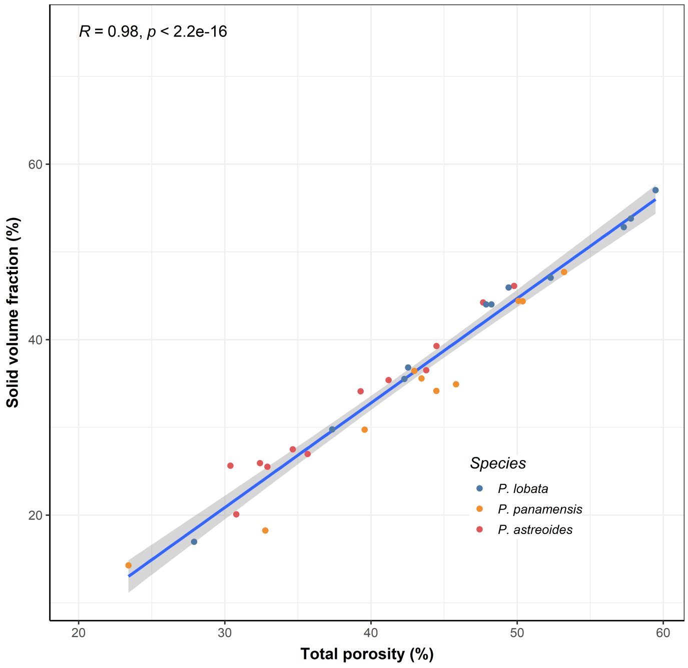

Figure S5. Slice (2D tomography representation) for control samples of rhodolites.

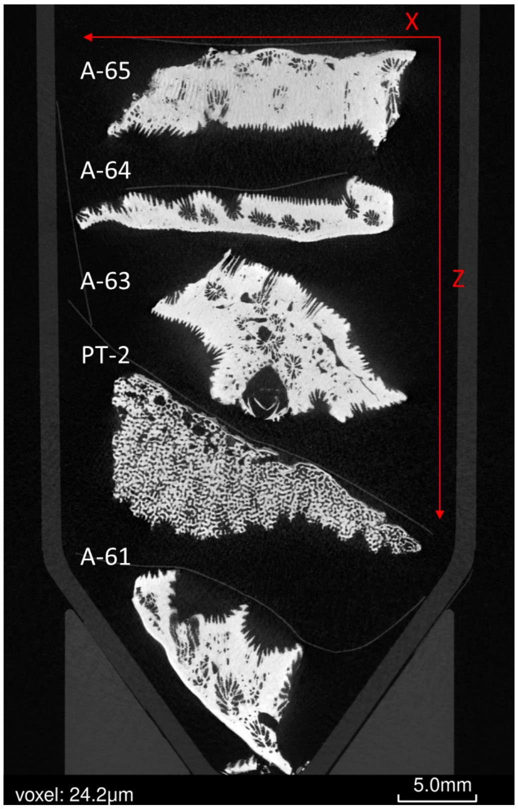

Figure S6. Control profiles of rhodolites for X,Y & Z offset coordinates. Even with some fluctuations, standard errors are commonly overlapped between coordinates.

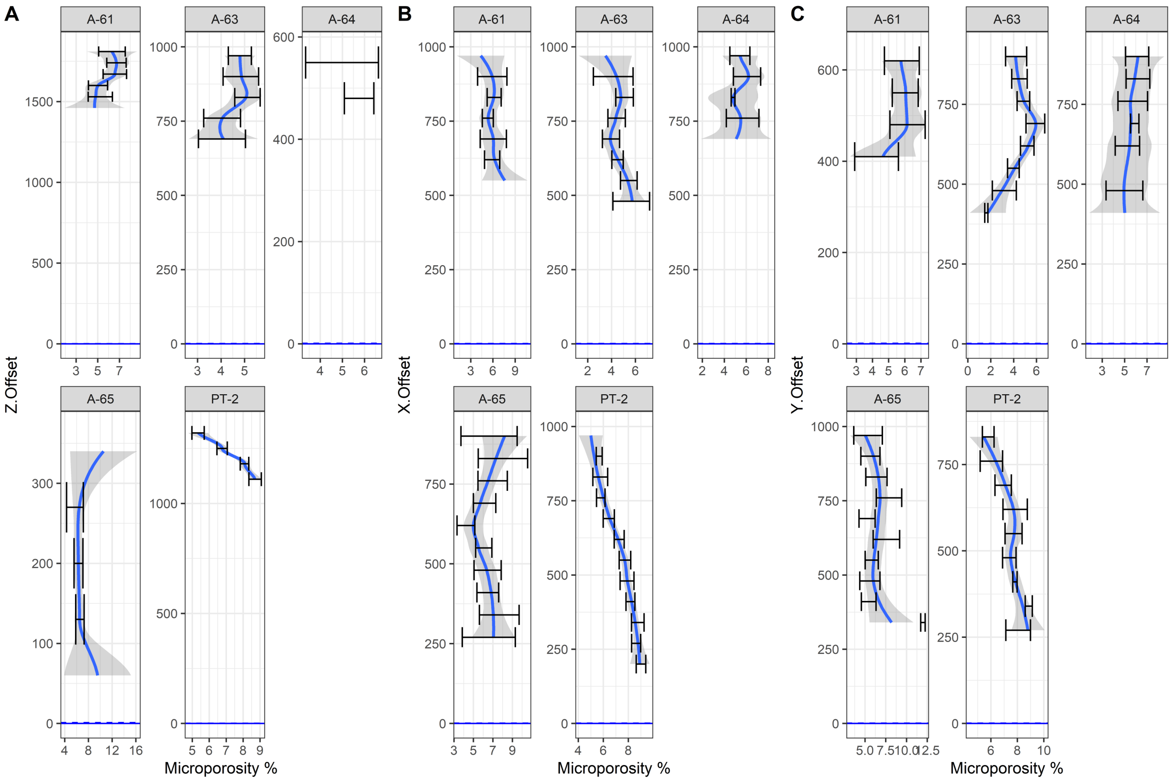

Figure S7. Pictures of the 12 selected coral fragments (Intra-colonial analysis).

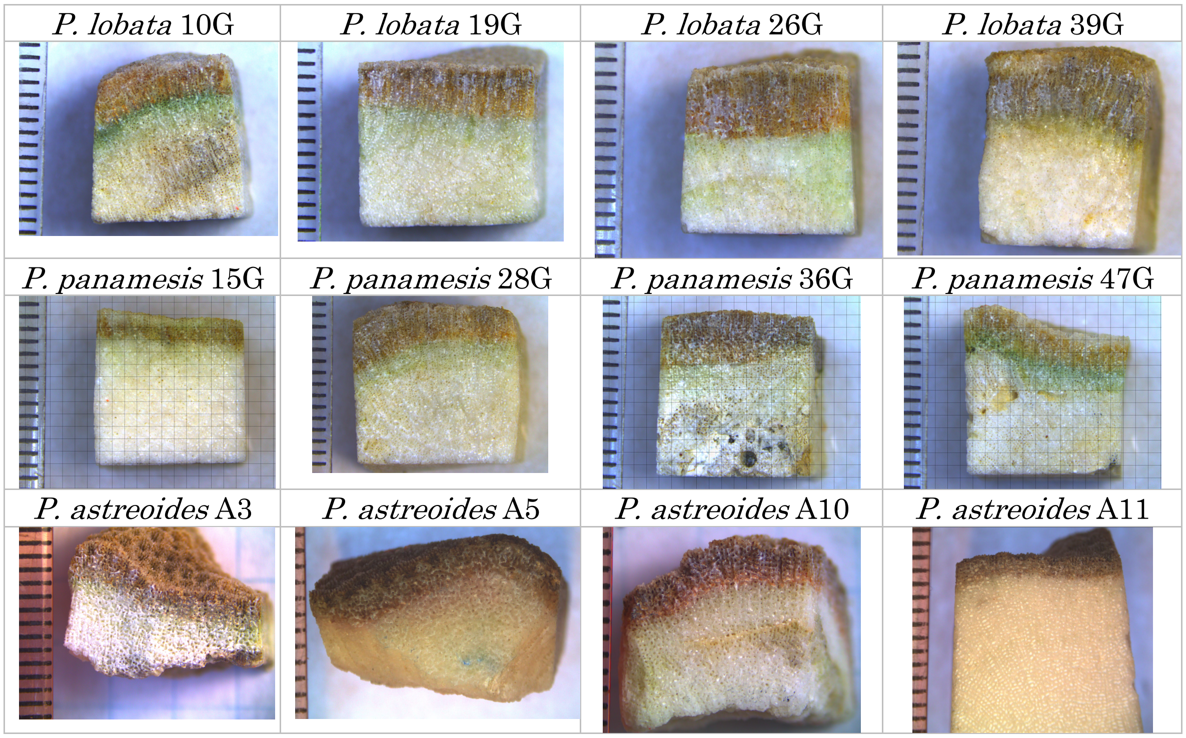

Supplement: Supplementary file 1 — Supplementary Material 1 [file 41598_2025_99374_MOESM1_ESM.pdf]
